# PD-1 Blockade Aggravates Epstein–Barr Virus^+^ Post-Transplant Lymphoproliferative Disorder in Humanized Mice Resulting in Central Nervous System Involvement and CD4^+^ T Cell Dysregulations

**DOI:** 10.3389/fonc.2020.614876

**Published:** 2021-01-12

**Authors:** Valery Volk, Sebastian J. Theobald, Simon Danisch, Sahamoddin Khailaie, Maja Kalbarczyk, Andreas Schneider, Julia Bialek-Waldmann, Nicole Krönke, Yun Deng, Britta Eiz-Vesper, Anna Christina Dragon, Constantin von Kaisenberg, Stefan Lienenklaus, Andre Bleich, James Keck, Michael Meyer-Hermann, Frank Klawonn, Wolfgang Hammerschmidt, Henri-Jacques Delecluse, Christian Münz, Friedrich Feuerhake, Renata Stripecke

**Affiliations:** ^1^ Laboratory of Regenerative Immune Therapies Applied, REBIRTH - Research Center for Translational Regenerative Medicine, Department of Hematology, Hemostasis, Oncology and Stem Cell Transplantation, Hannover Medical School, Hannover, Germany; ^2^ German Centre for Infection Research (DZIF), Partner site Hannover, Hannover, Germany; ^3^ Institute for Pathology, Hannover Medical School, Hannover, Germany; ^4^ Department of Systems Immunology, Braunschweig Integrated Centre of Systems Biology, Helmholtz Centre for Infection Research, Braunschweig, Germany; ^5^ Viral Immunobiology, Institute of Experimental Immunology, University of Zürich, Zürich, Switzerland; ^6^ Institute for Transfusion Medicine and Transplant Engineering, Hannover Medical School, Hannover, Germany; ^7^ Department of Obstetrics, Gynecology and Reproductive Medicine, Hannover Medical School, Hannover, Germany; ^8^ Institute for Laboratory Animal Science, Hannover Medical School, Hannover, Germany; ^9^ The Jackson Laboratory, Sacramento, CA, United States; ^10^ Biostatistics Group, Helmholtz Centre for Infection Research, Braunschweig, Germany; ^11^ Institute for Information Engineering, Ostfalia University, Wolfenbuettel, Germany; ^12^ Research Unit Gene Vectors, Helmholtz Zentrum München, German Research Center for Environmental Health and German Centre for Infection Research (DZIF), Partner site Munich, Munich, Germany; ^13^ German Cancer Research Center (DKFZ), Institut National de la Santé et de la Recherche Médicale (INSERM) Unit U1074, Heidelberg, Germany; ^14^ Institute for Neuropathology, University Clinic Freiburg, Freiburg, Germany

**Keywords:** humanized mice, immuno-oncology, Epstein-Barr Virus (EBV), immune checkpoint inhibition (ICI), post-transplant lymphoproliferative disease (PTLD), lymphoma, PD-1, pembrolizumab

## Abstract

Post-transplant lymphoproliferative disorder (PTLD) is one of the most common malignancies after solid organ or allogeneic stem cell transplantation. Most PTLD cases are B cell neoplasias carrying Epstein-Barr virus (EBV). A therapeutic approach is reduction of immunosuppression to allow T cells to develop and combat EBV. If this is not effective, approaches include immunotherapies such as monoclonal antibodies targeting CD20 and adoptive T cells. Immune checkpoint inhibition (ICI) to treat EBV^+^ PTLD was not established clinically due to the risks of organ rejection and graft-*versus*-host disease. Previously, blockade of the programmed death receptor (PD)-1 by a monoclonal antibody (mAb) during *ex vivo* infection of mononuclear cells with the EBV/M81^+^ strain showed lower xenografted lymphoma development in mice. Subsequently, fully humanized mice infected with the EBV/B95-8 strain and treated *in vivo* with a PD-1 blocking mAb showed aggravation of PTLD and lymphoma development. Here, we evaluated *vis-a-vis* in fully humanized mice after EBV/B95-8 or EBV/M81 infections the effects of a clinically used PD-1 blocker. Fifteen to 17 weeks after human CD34^+^ stem cell transplantation, Nod.Rag.Gamma mice were infected with two types of EBV laboratory strains expressing firefly luciferase. Dynamic optical imaging analyses showed systemic EBV infections and this triggered vigorous human CD8^+^ T cell expansion. Pembrolizumab administered from 2 to 5 weeks post-infections significantly aggravated EBV systemic spread and, for the M81 model, significantly increased the mortality of mice. ICI promoted Ki67^+^CD30^+^CD20^+^EBER^+^PD-L1^+^ PTLD with central nervous system (CNS) involvement, mirroring EBV^+^ CNS PTLD in humans. PD-1 blockade was associated with lower frequencies of circulating T cells in blood and with a profound collapse of CD4^+^ T cells in lymphatic tissues. Mice treated with pembrolizumab showed an escalation of exhausted T cells expressing TIM-3, and LAG-3 in tissues, higher levels of several human cytokines in plasma and high densities of FoxP3^+^ regulatory CD4^+^ and CD8^+^ T cells in the tumor microenvironment. We conclude that PD-1 blockade during acute EBV infections driving strong CD8^+^ T cell priming decompensates T cell development towards immunosuppression. Given the variety of preclinical models available, our models conferred a cautionary note indicating that PD-1 blockade aggravated the progression of EBV^+^ PTLD.

## Introduction

EBV is an oncogenic γ-herpesvirus associated with around 2% of all human cancers such as hematologic and epithelial malignancies (1). After the primary infection, EBV lytic reactivations are immunologically controlled, but the virus is not cleared. The life-long EBV maintenance in the host does not always lead to cellular transformations, but dysfunctional immunity can predispose opportunistic flares of EBV reactivations and eventually lead to EBV^+^ infectious mononucleosis (IM), lymphoproliferative diseases (LPD), or malignancies (2). Thus, novel immunotherapeutic options are being developed and tested for treatment of EBV infections and/or EBV-associated malignancies. For example, immune checkpoint inhibition (ICI) with monoclonal antibodies (mAbs) became a major breakthrough immunotherapy for cancer treatment over the past decade and showed to be effective against several types of tumors. Currently, several mAbs blocking the programmed death receptor protein (PD)-1 or its ligand PD-L1 have been approved or are in clinical development against several types of cancer (3, 4). ICI has demonstrated impressive high response rates as salvage therapy in relapsed/refractory (R/R) classical Hodgkin lymphoma (cHL) (5), the most common cancer among young people. Incidentally, EBV contributes to the chronic inflammatory microenvironment of a subset of cHL with high expression of PD-L1. Indeed, the interaction of PD-L1 with PD-1 on inflammatory T cells constitutes one of the major immune escape mechanisms in cHL, associated with development of dysfunctional and tolerogenic T cell responses (5). The preclinical observation of PD-L1 overexpression in cHL led to the evaluation of ICI administration in several clinical trials and the subsequent clinical approval of nivolumab, a mAb blocking PD-1, for cHL therapy (6). In 2017, The U.S. Food and Drug Administration also granted approval to pembrolizumab (KEYTRUDA®), another PD-1 blocking mAb, for the treatment of adult and pediatric patients with refractory cHL.

Another important clinical scenario is the EBV-associated post-transplant lymphoproliferative disorder (PTLD), a severe complication occurring in immunocompromised hosts after allogeneic hematopoietic stem cell transplantation (allo-HCT) or solid organ transplantation (SOT). The diagnostic of PTLD categories proposed by World Health Organization (WHO) are early lesions, polymorphic PTLD, monomorphic PTLD, and also cHL (7). Diffuse large B-cell lymphoma (DLBCL) accounts for the majority of the cases of PTLD. Development of aggressive EBV^+^ DLBCL has been associated with both latent and lytic viral cycles (8). The EBV^+^ DLBCL subtype is amonomorphic B cell lymphoma positive for EBV-encoded small RNAs (EBER) and characteristically expressing pan-B-cell markers (CD19, CD20, and occasionally CD30) with an EBV latency pattern type III in which all latency genes are expressed [for an expert review see (9)]. A small number of lytic infected cells can be found in tumors and show expression of lytic transcripts (such as BZLF1 and gp350) (8). A biomarker for these malignancies is the frequently detectable high expression of PD-L1 (CD274 and also known as B7-H1) in EBV^+^ PTLD, cHL, and DLBCL (10–12). PD-L1 induction in EBV^+^ malignancies occurs *via* AP-1 and JAK/STAT pathways (10) and also through the cooperation of EBV-LMP-1 and IFN-γ signaling (13). For EBV^+^ DLBCL, the PD-L1 positive tumors express high levels of indoleamine 2,3-dioxygenase (IDO), indicating a tolerogenic tumor microenvironment (TME) (11). Furthermore, EBV^+^ DLBCL presents with a distinct tolerogenic TME than EBV^−^ DLBCL, characterized by elevated expression of immune checkpoints [PD‐L1, PD‐L2, lymphocyte activation gene 3 (LAG-3) and T cell immunoglobulin and mucin domain-containing protein 3 (TIM-3)] and high levels of immunosuppressive M2‐type macrophages (12). Thirty to 40% of DLBCL patients show primary resistance or early relapse after standard therapy and there is no standard treatment approach specifically for aggressive EBV^+^ DLBCL (9).

The standard of care for PTLD/DLBCL is first a reduction in immunosuppression, that needs to be adjusted to the clinical situation, e.g. potential risks for transplant rejection or graft-*versus*-host disease (GvHD) (2). Additionally, treatment with rituximab, a mAb that targets the CD20^+^ malignant cells, produces response rates of about 65% (14). Clinical trials in specialized centers demonstrated that adoptive administration of third party or donor-derived or T cells specific for EBV could effectively restore virus-specific immunity and confer protection in 60 to 90% of transplant recipients with a PTLD (15). In addition to these measures, use of ICI could be a promising approach to treat EBV^+^ PTLD (10). Nonetheless, since the immunity of patients after transplantations is not in the normal homeostatic steady state, the clinical use of ICI against PTLD demands careful preclinical testing, particularly due to the associated risks of graft rejection and graft-versus host disease (GvHD).

EBV strictly infects human cells and therefore special *in vivo* models are required for preclinical testing of therapies targeting an EBV^+^ malignancy or immunopathology. During the last decade, mice reconstituted with the human immune system (“HIS mice”) became the state-of-the art *in vivo* models to test human-specific therapies against several human infections, malignancies, immunological and metabolic diseases (16–18). Infection of HIS mice with different EBV strains recapitulated several hallmarks of PTLD and lymphoma development and, concurrently, allowed studies of the human T cell inflammatory immune responses (19–22). The EBV/B95-8 strain was originally isolated from a patient with infectious mononucleosis and was later modified into the engineered B95-8/GFP strain, produced in the laboratory with the bac-mid technology. B95-8/GFP promotes a high B cell transformation rate and B-cell lymphoma growth (23). We have previously shown that, a few weeks after infection of HIS mice with B95-8/GFP, a dramatic expansion of CD8^+^ T cells expressing the inhibitory receptors PD-1, LAG-3, and TIM-3 followed (24, 25). Another strain, EBV/M81, was originally isolated from a patient with nasopharyngeal carcinoma (26). The M81/GFP engineered laboratory derivative induces potent lytic replication in B cells both *in vitro* and in humanized mice also leading to development of tumors (23).

Previously, therapeutic effects of PD-1 blockade in a xenograft mouse model of M81/GFP^+^ lymphoma were reported (27) (**Table 1**). Mononuclear cells obtained from cord blood (CB) were infected *in vitro w*ith the M81/GFP strain and then injected *i.p.* into Nod.Scid.Gamma (NSG) mice. Tumors resembling diffuse large B cells lymphoma (DLBCL) developed 3–5 weeks after in spleen. Pre-treatment of the cells with mAbs *in vitro*, i.e., prior to administration into the mice, significantly lowered the development of EBV^+^ tumors, particularly when anti-PD-1 and anti-CTLA-4 mAbs were combined (27). More recently, the Münz laboratory showed that fully humanized mice infected with B95-8/GFP and afterwards treated with a PD-1 blocking mAb showed significant increase in EBV viral load and tumor development (25) (**Table 1**). The interpretation of these results was that the disruption of the PD-1/PD-L1 axis during the T cell priming phase abrogated functionality of CD8^+^ CTLs, required to keep EBV infection and tumor development under control (25).

**Table 1:** Summary of experimental approaches and conclusions from three different animal studies evaluating *in vivo* the effects of PD-1 blockade on EBV infection and tumor development.

	Ma et al. (27)	Chatterjee et al. (25)	This Study
**Human Donor**	Newborn cord blood Commercial	Fetal liver tissue Commercial	Newborn cord blood On site collection
**Human Cells or Tissues**	CD34-depleted mononuclear cells. Dose: 12–25× 10e6 cells/mouse	HLA-A2^+^ CD34^+^ isolated HSCs Dose: 1–3 × 10e5 HSCs/mouse	CD34^+^ isolated HSC Dose: 2 × 10e5 cells/mouse
**Mouse Recipient**	NSG/Jackson Laboratory/3–5 weeks old at cell transfer	NSG w.t. or expressing HLA-A2/Jackson Laboratory/HCT in newborns up to 5 days of age	NRG*/*Breeding pairs Jackson Laboratory/HCT in 5–6 weeks old mice/Females
**Mouse Handling**	Human xenograft model/Intraperitoneal cell transfer after *in vitro* infection/Euthanasia at endpoint 4 weeks after cell transfer, or earlier if clinically ill	Fully humanized model/Sublethally irradiated (1 Gy)/intrahepatic HCT/EBV infection 12 weeks after HCT/Bleedings 2, 3, 4, 5 wpi/Euthanasia at endpoint 5 wpi, or earlier if ill	Fully humanized model/Sublethally irradiated (4.5 Gy)/IV HCT/EBV infection 15–17 weeks after HCT/Bleedings 0, 2, 3–4, 5–6, 8 wpi/Optical Imaging 2, 3–4, 5–6 wpi/Euthanasia at endpoint 8 wpi, or earlier if ill
**Human Immunity**	Spleen and tumors at endpoints. FACS: HLA-A/B/C, CD45, CD3, CD19, CD20, PD-L1, PD-L2, PD-1, CTLA-4	Blood FACS: CD45, CD19, CD3, CD4, CD8 Endpoint FACS CD3, CD8, CD19, CD27, CD28, CD45, CD45RA, CD62L, CTLA4, HLA-DR, Ki67 LAG-3, PD-1, PD-L1, Tim-3 and other analyses of human cytokines in plasma	Blood analyses FACS: CD45, CD19, CD3, CD4, CD8 Endpoint FACS CD3, CD8, CD19, CD45, PD-1, LAG-3, TIM-3 FoxP3 Analyses of human cytokines in plasma
**Human Infections**	EBV M81/GFP produced in HEK293/tittered on Raji cells/CB cells infected *in vitro* with 2,000 to 5,000 infectious units for 1.5 h	B95-8/GFP produced in HEK293/Tittered on Raji cells/EBV infection IP with 10e5 infectious units/Viral load: DNA isolated from spleen and whole blood and RT-qPCR	B95-8/fLuc/GFP and EBV M81/fLuc/GFP produced in HEK293/tittered on Raji cells/B95-8 10e5 and M81 10e6 infectious units IV/Followed for up 8 wpi/Viral load: DNA isolated from spleen and bone marrow and RT-qPCR
**Human Oncology**	Lymphoma development 4 weeks after cell transfer/spleen and organs H&E staining/IHC staining CD20, CD8, CD4/EBV antigens: EBER, EBNA2, LMP1, BZLF1, BMRF	PTLD and lymphoma terminal analyses 5 wpi/Spleen IHC EBNA1, EBNA2, EBER	PTLD and lymphoma terminal analyses 8 wpi/Spleen, liver, brain, IHC CD20, CD30, Ki67, CD3, CD4, CD8, PD-1, PD-L1, LMP1, EBNA2, EBER
**Pre-clinical Study and Outcomes**	No ICI *in vivo*/Anti-CTLA-4 mAb (Ipilimumab)/anti-PD-1 mAb (anti-hCD279, clone J116 BioXcell)/3× weekly (100 μg i.p.), starting 5–10 days after cell transfer until 4 weeks after cell transfer/Control huIgG ICI applied *in vitro* reduced lymphomas as single therapies (*P=0.06*) or combination (*P=0.03*)/combination, lowered # latently, and lyrically infected B cells, increased EBV-specific T cell responses **ICI considered useful for treating EBV-induced diseases**	ICI starting 3 wpi applied a total of 7× until 5 wpi/Anti-PD-1 mAb (clone EH12.2H7 Biolegend) 100–150 μg per administration/7× administrations/Control: PBS or 150 μg of isotype control antibody ICI applied during *in vivo* T cell priming resulted in elevated viral load (blood *P < 0.05*, spleen *P < 0.001*), elevated tumor burden (*P < 0.05*./CD3 T cell numbers in blood not changed/Lower frequencies of PD-1^+^ CD8 T cells (*P < 0.0001*)/Elevated levels of cytokines (IFN-γ, TNF-α, IL-10, IL-8 *P < 0.05* to *P < 0.001*) **Function of PD-1 axis necessary for control of EBV infection and tumorigenesis**	ICI low dose: 1^st^ administration 3.33 mg/kg at 2 wpi; 1.7 mg/kg every other week applied 6×)/ICI high dose (1^st^ 10 mg/kg at 2 wpi; 5 mg/kg every other week applied 6×) ICI applied during *in vivo* T cell priming resulted in elevated viral spread (BLI) load (spleen and bone marrow), elevated tumor burden (also in CNS) and higher mortality/CD4 and CD8 T cell numbers in blood reduced, lower frequencies of PD-1^+^ CD8 and CD4 T cells/Increased frequencies of TIM-3^+^, LAG-3^+^ and Tregs/elevated levels of cytokines (IFN-α, IL-10, IL-33, IL-12) **PD-1 blockade worsens PTLD and lymphomagenesis with the emergence of immune dysfunctional T cells**

In view of these opposite results, we sought to clarify these discrepancies in fully humanized mice comparing head-to-head the two EBV strains used for infection models (**Table 1**). NOD.Rag.Gamma (NRG) HIS mice were infected either with EBV/B95-8/fLuc or EBV/M81/fLuc and dynamic non-invasive monitoring of viral spread and post-mortem immunological and histological analyses were performed as previously reported (24, 28). We show a dose-dependent effect of pembrolizumab augmenting the EBV systemic dissemination, lymphomagenesis, and increasing mortality. Remarkably, pembrolizumab treatment promoted EBV spread into the central nervous system (CNS) recapitulating CNS-PTLD. These severe effects of PD-1 blockade were associated with a decompensation of the systemic and intra-tumoral pattern of CD4^+^ T cells and accumulation of cytokines and FoxP3^+^ T cells. Thus, although PD-1 blockade may be a favorable therapeutic option against some EBV^+^ tumors, these models show that it can aggravate the EBV infections in the context of PTLD, promoting hyper-progressive disease (HPD), morbidity, and mortality.

## Material and Methods

### Generation of Humanized NRG Mice

Collection of CB was performed at the Department of Gynecology and Obstetrics (Hannover Medical School) and obtained according to study protocols approved by the Ethics Committee of the Hannover Medical School and after informed consent obtained from the mothers. Human CD34^+^ hematopoietic cells were isolated from CB after two rounds of positive selection using immune magnetic beads (Direct CD34 Progenitor Cell Isolation Kit, human, MACS Miltenyi Biotec, Bergisch Gladbach, Germany) as described (24, 28–30). All experiments involving mice were approved by the Lower Saxony Office for Consumer Protection and Food Safety–LAVES (permit number: 16/2222) and performed in accordance with the German animal welfare act and the EU-directive 2010/63. Breeding pairs of $\text{NOD.Cg}-Rag1^{tm1Mom}IL-2R\gamma_{c}^{tm1Wjl}$ (NRG) mice were obtained from the Jackson Laboratory (JAX; Bar Harbor, USA) and bred under pathogen-free conditions. Prior to HCT, 5–6 weeks-old mice were sublethally irradiated (450 cGy) using a [^137^Cs] column irradiator (Gammacell 3000 Elan; Best Theratronics, Ottawa, Canada). Four hours after irradiation, 2.0 × 10^5^ CD34^+^ cells were injected through the tail vein. Body weight and occurrence of GvHD were monitored weekly after HCT. For the sake of reproducibility, we used preferentially female NRG mice because they show more consistent and stable human immune reconstitution and immune responses >15 weeks after HCT (29). Further, CD34^+^ HSC units were pretested in a couple of transplanted NRG mice and only those resulting in 20% or higher frequencies of human CD45^+^ cells in mouse blood at 10–15 weeks post-HCT were used for experiments with EBV infections. These parameters were extensively optimized for preclinical testing of vaccines and chimeric antigen receptor (CAR) T cell therapies (28, 30–33).

### EBV Infections and Treatment of Humanized Mice With Pembrolizumab

Stable human hematopoietic engraftment was confirmed in peripheral blood at 15–17 weeks after HCT, and at this point mice were infected with the B95-8/fLuc (24) or with the M81/fLuc strain (28, 34). The engineered B95-8/fLuc strain expresses the reporter gene firefly luciferase downstream of the viral Epstein-Barr nuclear antigen 2 (EBNA2) (24), while the EBV/M81/fLuc strain expresses fLuc under the control of an inserted CMV immediately early (IE)1 promoter sequence (34). EBV titers were defined as green Raji units (GRU) after infection of Raji cells with serial dilutions of the virus stocks. The percentage of GFP^+^ cells was analyzed by flow cytometry 3 days later and the viral titers were calculated by the following formula: “total number of Raji cells infected” × “percentage of GFP^+^ cells”/”volume of the virus stock used.” After pilot experiments to evaluate the relationship between the viral dose and reproducible bioluminescent signals detectable at 2 weeks post infection (wpi), the viral doses diluted in 100 µl PBS were established: 10^5^ GRU for EBV/B95-8/fLuc and 10^6^ GRU for EBV/M81/fLuc (**Table 1**). Using these viral doses, we have reported that most of the infected HIS mice survive for more than 8 wpi with some mice showing progressive disease while others are able to control the tumor development with stable disease (24, 28). Euthanasia was performed at the endpoint 8 wpi for collection of biopsies or earlier if symptoms of distress due to tumor development or weight loss were detected. At 2 wpi, mice were randomized based on their bioluminescence signal between PBS control, pembrolizumab high dose (HD) and pembrolizumab low dose (LD). Pembrolizumab (MSD; Kenilworth; USA) was diluted in PBS for administration as a low dose (LD: First administration 3.33 mg/kg at 2 wpi; 1.7 mg/kg every other week applied six times) or as a high dose (HD: First administration 10 mg/kg at 2 wpi; 5 mg/kg every other week applied six times). As treatment controls PBS or human pooled immunoglobulin (KIOVIG, a clinically used pooled polyclonal IgG product obtained from human donors) were injected intraperitoneally every week after 2 wpi.

### Flow Cytometry Analysis of Blood and Lymphatic Tissues

Peripheral blood (BL) obtained at different time-points and tissues obtained from humanized mice at the endpoint [lymph node (LN), spleen (SPL), bone marrow (BM), thymus (Thy)] were processed as previously described (24, 29, 30). For BL and SPL, a hypotonic solution (0.83% ammonium chloride/20 mM HEPES, pH 7.2, for 5 min at room temperature) was used to remove erythrocytes. Flow cytometry acquisitions were performed with fresh or cryopreserved/thawed cells after immune staining (see antibodies used in **Table S10**). After washing of unbound antibodies, cells were acquired with an LSR II cytometer (BD bioscience) or Cytoflex cytometer (Beckman Coulter). Data was analyzed with FlowJo (LLC, USA).

### Dynamic Bioluminescence Imaging (BLI) Analysis

Viral infection and spread in the mice were monitored by expression of luciferase in tissues infected with EBV/B95-8/fLuc or with EBV/M81/fLuc. Dynamic BLI analyses were performed with the IVIS SpectrumCT apparatus (PerkinElmer, Waltham, MA, USA). Before imaging, mice were anesthetized using isoflurane. Mice were imaged 5 min after luciferin administration [2.5 mg D-luciferin potassium salt intraperitoneally (SYNCHEM, ELK Grove Village, IL, USA) reconstituted in 100 µl PBS]. Data was analyzed using the Living Image Software (PerkinElmer, Waltham, MA, USA).

### Quantification of Epstein–Barr Virus by Real-Time Quantitative PCR

DNA was isolated from tissues using the DNeasy Blood&Tissue Kit (QIAGEN, Venlo, Netherlands) following the manufacturer´s protocol. In order to detect EBV DNA, a quantitative PCR (qPCR) was performed by amplifying a fragment of the BALF5 gene using following primers: 5´-CTTTGGCGCGGATCCTC-3´ (forward) and 5´-AGTCCTTCTTGGCTAGTCTGTTGAC-3´ (reverse). Detection of the amplification was performed with the following Taqman probe: 5´-Fam-CATCAAGAAGCTGCTGGCGGCC-Tamra-3´. EBV copy number was normalized per µg of isolated DNA. The Taqman PCR assays were run in duplicates on the StepOnePlusTM Real-time system (Applied Biosystems, Life Technologies, Darmstadt, Germany). Data was analyzed using the StepOnePlusTM software (Applied Biosystems, Life Technologies, Version 2.3).

### Cytokine Analysis in Mouse Plasma

Cytokine concentrations in plasma samples were determined using the LEDGENDplex™ Human Inflammation Panel 1 Multi-Analyte Flow Assay (BioLegend, San Diego, USA), which allowed for the simultaneous detection of IFN-α, IFN-γ, IL-1β, IL-6, IL-8, IL-10, IL-12p70, IL-17A, IL-18, IL-23, IL-33, MCP-1, and TNF-α. The assay was performed according to the manufacturer’s instructions. Briefly, undiluted samples and standards were incubated with antibody-coated premixed beads overnight at 4°C followed by incubation with detection antibody for 1 h at room temperature and streptavidin-PE for 30 min at room temperature. Samples were read on a BD FACSCanto™ Flow Cytometer (BD Biosciences, Heidelberg, Germany) and analyzed with the LEGENDplex v8.0 Software (BioLegend, San Diego, USA).

### Histopathology, Immunohistochemistry, and Image Analysis

Three-µm-thick tissue sections were cut from formalin-fixed, paraffin-embedded (FFPE) tissue blocks containing mouse SPL and LI. Staining was performed according to standard protocols for Giemsa. Immunohistochemistry analyses were performed using the Benchmark Ultra automated instrument (Ventana/Roche Tissue Diagnostics, Mannheim, Germany). Following antibodies were used for tissue staining: Ki67 (clone RM9106-S1; ThermoScientific), CD20 (clone M0755; Dako), CD30 (Ber-H2; Dako), CD3 (clone M7254; Dako), programmed cell death ligand 1 (PD-L1; clone 22C3; Dako), CD8 (clone C8/144B; Dako, Copenhagen, Denmark), CD4 (clone SP35; Zytomed Systems, Berlin, Germany), PD-1 (MRQ-22; Medac Diagnostika, Wedel, Germany), FoxP3 (cat. number 560046; BD) (vendor information available in **Table S10**). For antibody binding detection, the 3,3-diaminobenzidine based UltraView reagent was used according to the manufacturer’s recommendations. EBER-1 detection was performed by an *in situ* hybridization method (EBER 1 DNP Probe, Ventana/Roche Tissue Diagnostics) and the automated Benchmark Ultra instrument. Whole Slide Scans were produced using an Aperio AT2 scanner (Leica Biosystems), images were taken using the ImageScope software, and trained pathologists evaluated staining results.

### Quantitative Multispectral Immunohistochemistry

Three-µm-thick FFPE tissue slides were deparaffinized and antigen retrieval was performed according to “Opal Multiplex IHC Assay Development Guide” instructions. Blocking was done using Protein Block Serum-free (Dako) solution before each staining cycle. Staining panel was designed to include primary antibodies against human CD4, CD8, FoxP3 (or CD30 for brain tissue staining), CD20, and Ki67. Secondary HRP Labelled Polymer mouse/rabbit (Dako EnVision+ System- HRP Labelled Polymer) antibodies were applied after washing step and maintained for 10 min at room temperature, followed by 10 min incubation with tyramide fluorophores (Opal 520, Opal 570, Opal 620, Opal 650 or Opal 690, Akoya) used for signal amplification. DAPI (Akoya) was used for nuclear staining and Fluoromount-G mounting medium (ThermoScientific) was applied to cover slides before imaging. Whole slide tissue scanning was done at 40× magnification using the “Vectra Polaris” (Akoya) platform. Spectral libraries were generated using single stained scans of tonsil tissue for each marker and images deconvolution was done with the inForm Advanced Image Analysis software (inForm v2.4.8; Akoya). Several representative spleen and liver images were used to train pre-designed machine learning algorithms within inForm software to define tissue classes (tumor, parenchyma, and blank area), adjust cell segmentation settings, and phenotype cells. All the settings from the training images were applied for batch analysis of scanned images.

### Statistical Analyses

Log-rank (Mantel-Cox) test was used to compare survival curves. Fisher’s exact test was applied to compare the number of macroscopically visible tumors between treatment groups. Unpaired Welch’s t test was used to calculate statistical significance for kinetics of immune reconstitution, EBV and T cell levels in tissues. Mann-Whitney t test was used for analyses of cytokines concentrations. P < 0.05 was considered statistically significant. Log-transformation of absolute numbers was done for BLI, RT-qPCR, PD1/CD4, and PD1/CD8 MFI, and cell count results, to compare the distribution of measurements within different groups; we also tested the equality of one-dimensional probability distributions using a two-sample Kolmogorov-Smirnov (KS) test. The KS statistic (D), defined as the maximal vertical distance between the empirical cumulative distributions between the groups, and significance level (p-value) were calculated and used as two criteria for finding biomarkers with significant difference between experimental groups. The biomarkers with high KS statistics and low p-value are reported. Statistical analyses were performed using GraphPad Prism v7 software (GraphPad Software Inc., La Jolla, CA, USA). KS test was performed using the statistical computing software R (version 3.4.3).

### Data Analyses

Quantitative imaging analyses of data tables exported from inForm 2.4.8. (Akoya) were performed using “R” packages “Phenoptr” and “PhenoptrReports” (Kent S Johnson (2019). phenoptr: inForm Helper Functions. R package version 0.2.3.). Analyses were performed using Rstudio (Rstudio v1.1.456). See also: https://www.r-project.org/foundation/. The readout protocols combined results for “Cell absolute counts,” “Cell percentages,” “Cell densities,” and “Counts within” following manufacturer’s instructions (Phenoptics™, Akoya).

## Results

### PD-1 Blockade in Fully Humanized Mice Promoted Epstein–Barr Virus Spread, Tumor Development, and Mortality

**Figure 1:**
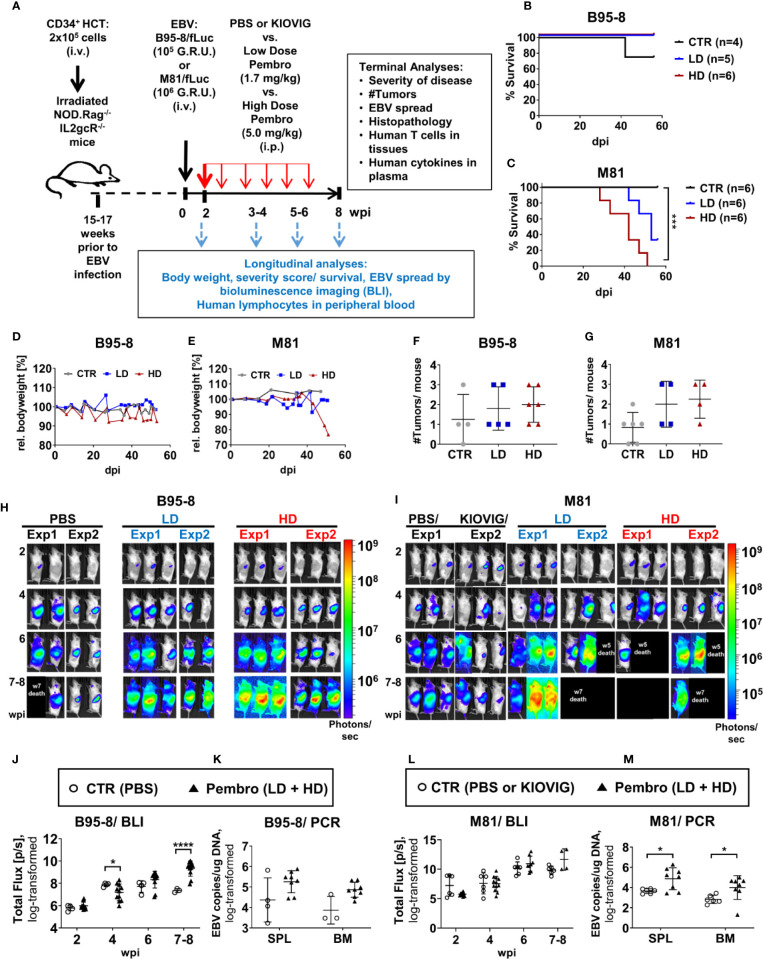
Humanized mice infected with EBV/fLuc and treated with pembrolizumab show high mortality associated with increased EBV spread and tumor development. **(A)** Scheme of the experiments. 2 × 10^5^ human CD34^+^ cord blood (CB) purified hematopoietic stem cells (HSCs) were used for hematopoietic stem cell transplantation (HCT) of sub-lethally irradiated NRG mice. Fifteen to 17 weeks after HCT, the long-term human immune reconstitution in blood (huCD45^+^) was confirmed. At this point, mice were infected i.v., with the preferentially latent EBV B95-8/fLuc strain (10^5^ GRU) or with the lytic EBV M81/fLuc strain (10^6^ GRU). Two independent experiments using HSCs from two different CB donors were performed for each EBV model. At 2 weeks post-infections (wpi), bioluminescence imaging (BLI) analyses were performed to determine the baseline of EBV infection level and to randomize the mice among three cohorts: (i) injected i.p. with phosphate-buffered saline (PBS, corresponding to the control group, CTR), (ii) injected i.p. a low dose (LD: First administration 3.33 mg/kg at 2 wpi; 1.7 mg/kg every other week applied six times), or with (iii) a high dose (HD: First administration 10 mg/kg at 2 wpi; 5 mg/kg every other week applied six times). Mice were monitored for disease severity every 2–3 days and body weights were measured weekly. Scores of disease severity or loss of 20% of body weight were used as termination criteria. Optical imaging analyses and analyses of human immune reconstitution in peripheral blood collections were performed longitudinally (2, 4, 6, 8 wpi). The experimental endpoint was eight weeks post-infections. The following data was acquired for terminal analyses: % survival, occurrence of weight loss, occurrence of tumors, EBV load, histopathology, characterization of T cells in blood and tissues, human cytokine profile in plasma. **(B, C)** Survival curves for humanized mice infected with the B95-8/fLuc strain **(B)** or with the M81/fLuc strain **(C)**. A log-rank test (Mantel-Cox) was applied to evaluate the differences in survival. Cohorts: CTR (gray line), LD (blue line); HD (red line). **(D, E)** Measurements of body weight relative to the baseline weights on the day of EBV infection. Mice infected with B95-8/fLuc strain **(D)** or M81/fLuc strain **(E)**. Cohorts: CTR (grea line), LD (blue line); HD (red line). **(F, G)** Numbers of macroscopically detectable tumors in mice infected with B95-8/fLuc **(F)** or with M81/fLuc **(G)**. Cohorts: CTR (gray dots), LD (blue dots); HD (red dots). **(H, I)** BLI pictures generated sequentially from week 2 to 8 after EBV infection performed in duplicate experiments for each EBV model and treatment. Pictures were taken of left body side of mice infected with B95-8/fLuc **(H)** or with M81/fLuc **(I)**. Bioluminescence signal intensities (photons/sec) are depicted by the color bars on the right side. Black boxes depict dead mice. **(J–L)** Quantification of EBV spread by BLI for the B95-8/fLuc **(J)** or M81/fLuc **(L)** strain. *Total Flux* corresponds to the radiance (photons/sec) in each pixel summed over the regions of interest (ROI) area containing the whole left side of the body. The dots represent quantifications at 2, 4, 6, 8 wpi for each mouse in CTR (open circles) or in pembrolizumab treatment cohorts (filled triangles, LD and HD were combined). **(K–M)** Quantification of EBV spread by RT-qPCR. EBV copies per µg DNA were quantified in spleen (SPL) and bone marrow (BM) of mice infected with B95-8/fLuc **(K)** or with M81/fLuc **(M)**. The dots represent quantifications for each mouse in CTR (open circles, PBS and KIOVIG controls combined) or in pembrolizumab treatment cohort (filled triangles, LD and HD were combined). Results of measurements were log-transformed and statistical analyses were performed using unpaired t test with Welch’s correction. Standard deviation is indicated. Statistical significances are indicated with *P < 0.05, ****P < 0.0001.

Pembrolizumab administration (“Pembro”) or the control treatment (“CTR”, PBS or KIOVIG) were initiated at 2 wpi, after a baseline BLI measurement was performed in order to allocate mice in comparable treatment arms (**Figure 1A**). The pembrolizumab low and high dose were previously determined in studies of humanized mice challenged with breast cancer and treated with the drug (35). EBV-infected mice were monitored for clinical signs of disease and euthanized based on humane endpoint criteria. BLI analyses were performed serially until 8 wpi. All B95-8/fLuc-infected mice treated with ICI survived until the end of the experiments (**Figure 1B**). In contrast, several mice infected with M81/fLuc and ICI-treated died prematurely and could not be analyzed or were euthanized (3/6 mice in the LD and in 6/6 in the HD cohort (survival of CTR *vs* HD, *P < 0.01*) (**Figure 1C**, **Table S1**). Mice infected with B95-8/fLuc treated with pembrolizumab or PBS showed comparable body weights (**Figure 1D**), while mice infected with M81/fLuc treated with HD pembrolizumab showed weight loss compared with the other arms (**Figure 1E**). For control infected mice, tumors typically developed in the spleen, while most ICI-treated mice showed multiple tumors located in different organs, such as liver, lung, and kidney (**Figures 1F, G**). At the baseline analyses before ICI treatment (2 wpi), EBV infection levels assessed by BLI analyses were comparable, and over time showed dynamic changes in intensities and bio-distribution. Remarkably, the BLI signals in the ICI-treated animals increased dramatically after 6–8 wpi indicating higher EBV dissemination (8 wpi: B95-8: CTR vs Pembro, *P < 0.0001*; M81: CTR vs Pembro, *P = 0.1387*) (**Figures 1H, I, J, L**, **Table 2**, **Table S2**). The EBV copy numbers in SPL and BM were higher in ICI-treated mice infected with B95-8/fLuc (**Figure 1K**, **Table 2**, **Table S3**, *P = 0.199* and *P = 0.111*, respectively) or with M81/fLuc than in controls (**Figure 1M**, **Table 2**, **Table S3**, *P < 0.05)*. In summary, pembrolizumab administration caused higher viral spread, viral load, tumor development for both EBV infection models. The M81/fLuc lytic model showed the highest severity leading to early mortality occurrences.

**Table 2:** Summary of EBV spread (by BLI and PCR) and immunologic effects (by FACS, cytokine measurement of plasma and histopathology analyses by multiplex immunohistochemistry) observed after PD-1 blockade.

A) B95-8	Endpoint wpi	Control			Pembrolizumab				
Analyses		N =	Mean	ST Dev	N =	Mean		ST Dev	P values
BLI p/s*	8	3	7.35	0.17	11	9.32		0.68	***<0.0001***
PCR BM copy/µg DNA*	8	3	3.87	0.67	8	4.87		0.36	0.1110
CD4-PD1 MFI PBL*	8	3	4.38	0.14	11	3.36		0.24	***0.0001***
CD4-PD1 MFI SPL*	8	3	4.94	0.24	11	3.50		0.29	***0.0013***
% Treg inCD4. SPL	8	3	2.80	1.80	11	6.50		5.70	*0.0876*
IL-17A pg/ml	8	3	1.63	0.93	10	8.87		9.81	***0.0490***
IL-10 pg/ml	8	3	416.73	681.52	10	825.43		718.53	0.2867
IL-33 pg/ml	8	3	3.90	0.48	10	10.57		17.06	0.1608
IFN-α pg/ml	8	3	3.07	3.15	10	11.39		12.09	*0.0769*
IL-12 pg/ml	8	3	10.29	16.37	10	3.56		2.79	0.5734
IL-1β pg/ml	8	3	1.89	0.52	10	2.89		2.59	0.8357
Treg near CD4 LI	8	3	0.02	0.023	11	0.35		0.40	***0.0193***
Treg near CD8 LI	8	3	0.001	0.02	11	0.25		0.30	***0.0251***
**B) M81**	**Endpoint** **wpi**	**Control**			**Pembrolizumab**				
**Analyses**		**N =**	**Mean**	**ST Dev**	**N =**	**Mean**	**ST Dev**		**P values**
BLI p/s*	8	6	9.84	0.68	4	11.65	1.82		0.1387
PCR BM copy/µg DNA*	5, 7, 8	6	2.83	0.41	8	4.00	1.18		***0.0283***
CD4-PD1 MFI PBL*	7, 8	6	4.84	0.08	5	3.96	0.41		***0.0074***
CD4-PD1 MFI SPL*	7, 8	6	5.15	0.06	5	3.99	0.59		***0.0112***
% Treg in CD4, SPL	7, 8	6	2.80	0.60	5	5.70	7.60		***0.0407***
IL-17A pg/ml	7, 8	6	3.71	1.73	5	4.07	4.04		0.6623
IL-10 pg/ml	7, 8	6	18.23	10.03	5	9189.12	11248.73		***0.0173***
IL-33 pg/ml	7, 8	6	3.35	1.03	5	51.80	70.27		***0.0087***
IFN-α pg/ml	7, 8	6	0.97	0.20	5	25.13	22.94		***0.0043***
IL-12 pg/ml	7, 8	6	1.51	0.51	5	59.21	105.47		***0.0043***
IL-1β pg/ml	7, 8	6	1.72	0.46	5	3.84	2.12		***0.0498***

(A) B95-8 infection. (B) M81 infection. The statistical methods used for analyses are shown in the **Supplementary Tables**. Significant differences between control and pembrolizumab-treated mice are shown in bold and italics. The original values for PD-1 MFI (*) were log-transformed before statistical tests. The reduced detections of PD-1 on CD4^+^ T cells were highly significant for both models (p values are underlined). The frequencies of CD4^+^ Tregs in spleens were increased for both models. The proximity of Tregs to T cells of liver for the B95-8 model was analyzed by multiplex immunohistochemistry analyses. The cytokine responses were variable for the two models.

### PD-1 Blockade Promotes Development of Larger Epstein–Barr Virus^+^ PD-L1^+^ Tumor Masses With Lower Densities of Tumor-Infiltrating Lymphocytes

**Figure 2:**
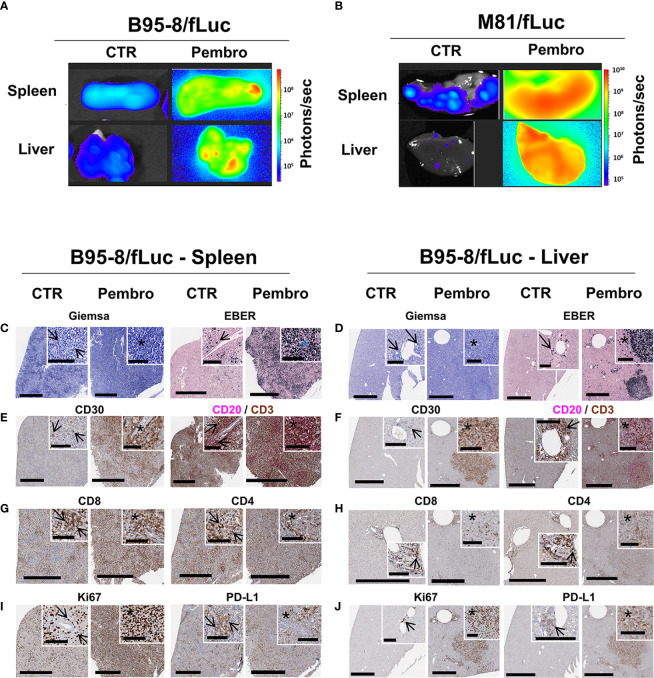
Analyses of explanted spleen and liver showed increased EBV/fLuc spread, inflammation, and neoplasia after pembrolizumab treatment. **(A, B)** Bioluminescence imaging analyses performed with explanted spleen and liver from representative B95-8/fLuc **(A)** or from representative M81/fLuc**-**infected mice **(B)**. Tissues from representative non-treated control (CTR) mice are shown. Bioluminescence signal intensities (photons/sec) are depicted by the color bars on the right side. **(C–J)** Histopathological analyses of spleen **(C–I)** and liver **(D–J)** of representative mice infected with EBV/B95-8/fLuc and non-treated (CTR, left side) or treated with pembrolizumab (Pembro, right side). **(C, D)** Giemsa, EBER: In both organs, the perivascular spread of EBV/B95-8/fLuc infected neoplastic cells is observed. More disseminated infection and larger tumors were observed in pembrolizumab-treated than in control mice. Stars indicate perivascular angiocentric neoplastic growth. **(E, F)** CD30, CD20/CD3 duplex: The CD30- and CD20-expressing tumors and perivascular infiltrates resemble human B-cell neoplasia, corresponding to diffuse large B-cell lymphoma, and are surrounded or invaded by variable amounts of tumor-infiltrating CD3^+^ lymphocytes (TILs). **(G, H)** CD8, CD4: Early perivascular lesions display a largely balanced CD4/CD8 ratio. In the center of tumor bulk mass, the balance is shifted towards CD8^+^ cytotoxic T lymphocytes (CTLs). **(I, J)** Ki67, PD-L1: proliferating cells observed at a higher density in tumor areas, which also correlates with PD-L1 expression. Digital whole slides scanned at 40×; black bars correspond to 600 µm (full image) and 100 µm (inserts).

Next, we dissected the effects of pembrolizumab at the tissue level and analyzed the histological signatures of the tumor formation. Post-mortem BLI analyses of explanted SPL and LI showed high levels of bioluminescence signals in ICI-treated mice (**Figures 2A, B**). Histopathological analyses confirmed numerous EBV^+^ lymphoid neoplastic lesions with a blast-like phenotype and a CD20^+^ and partially CD30^+^ immunophenotype of different size and cellular composition. The lesions ranged from relatively small, diffusely infiltrating or sheet-like tumor formations, often observed in perivascular localization (**Figures 2C–F**, left panels, arrows), to confluent lesions and massive compact tumor masses (**Figures 2C–F**, right panels, see areas marked with asterisks). In LI, large coherent tumor formations were almost exclusively observed in pembrolizumab treatment groups, while in control animals tumors tended to be smaller and only rarely confluent. In ICI-treated mice, large, partially necrotic tumor masses in SPL (**Figures 2C–E**, left panel) and LI (**Figures 2C–E**, right panels) were consistently present. The neoplastic partially CD30^+^ and CD20^+^ cells were associated with a dense CD3^+^ T-lymphocytic infiltrate (**Figures 2E, F**). Immune subtyping revealed that this T cell infiltrate was mainly composed of CD8^+^ cytotoxic T lymphocytes (CTLs) and CD4^+^ T helper cells (Th) (**Figures 2G, H**). Brisk T cell infiltrates were predominantly observed in small, presumably early-stage perivascular lesions (**Figures 2C–H**, arrows), where fewer neoplastic cells expressing EBER were present in relation to the overall amount of cells, and at the margins of coherent tumor masses. In contrast, the center of larger tumor formations (**Figures 2C–H**, see areas marked with asterisks) showed relatively fewer T cells, particularly fewer CD4^+^ Th cells, if compared to tumor margins and smaller perivascular tumor cell groups (**Figures 2G, H**). The tumor areas corresponded to proliferative activity: Ki67^+^ cells with morphological features indicative of neoplastic lymphoproliferation were more prominent in tissues of ICI-treated mice and tumor formations were associated with higher expression of PD-L1 (**Figures 2I, J**). Altogether, these descriptive analyses indicated that infiltrating T lymphocytes were more pronounced, and potentially more active, in early stages of tumor invasion and at the invasive edges, but less prominent in the center of the large tumor masses observed in ICI-treated mice, a pattern resembling “immune excluded” tumors (36).

### PD-1 Blockade Promotes Epstein–Barr Virus Spread into the Central Nervous System

**Figure 3:**
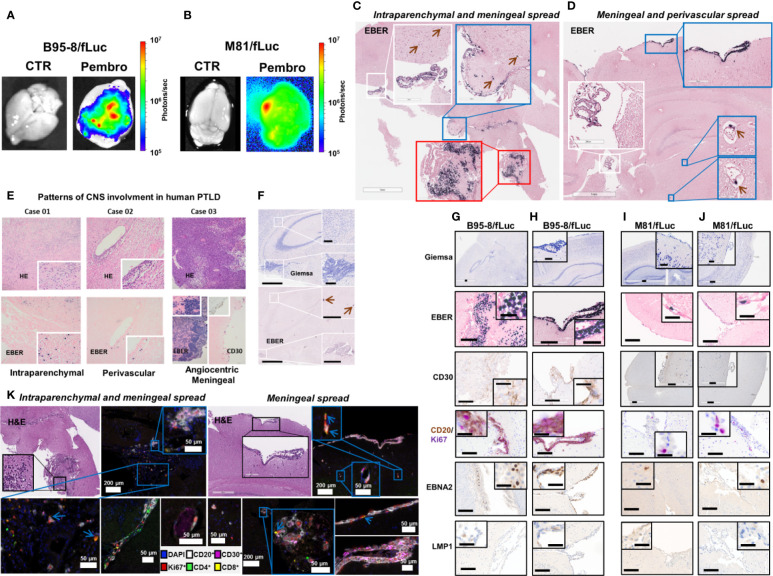
Pembrolizumab treatment is associated with spread of EBV/fLuc to the central nervous system. **(A, B)** Bioluminescence images showing representative EBV signal patterns in brains from mice infected with B95-8/fLuc **(A)** or with M81/fLuc **(B)** and treated with pembrolizumab (Pembro; most severely affected animals shown in each group), compared with non-treated controls (CTR). The bioluminescence signal intensities (photons/sec) are depicted by the color bar on the right side. **(C, D)** *In-situ* hybridization for detection of Epstein-Barr encoded RNA (EBER)-1 in brains of mice infected with B95-8/fLuc and treated with pembrolizumab. Observed patterns include invasion of tumor cells into the plexus choroideus (white inserts), as well as into periventricular brain parenchyma (red insert) and meningeal tumor spread (blue inserts). The green arrows indicate single tumor cells associated with blood vessels. **(E)** Comparison with three representative examples of human EBV-positive monomorphic transplantation-associated B-PTLD, with clinical manifestation as primary CNS lymphoma (PCNSL) revealed similar patterns of intraparenchymal (Case 01), perivascular (Case 02), and massive angiocentric/diffuse growth with meningeal spread (Case 03). **(F)** Control animals did not show any involvement of the plexus choroideus or periventricular brain areas. Only single EBER-positive cells in intravascular localization were detected (green arrows). **(G–J)** The immunophenotype of the neoplastic cells invading the brain of ICI-treated mice corresponded to the phenotype of the peripheral neoplastic population with variable CD30^+^, and constant CD20^+^ expression. The majority of neoplastic cells expressed EBNA2 but not LMP1 and were actively proliferating (CD20^+^/Ki67^+^). **(G–H)** and **(I–J)** Analyses of duplicate mice infected with B95-8/fLuc and M81/fLuc and treated with pembrolizumab, respectively. **(K)** Multiplexed image of two mouse brains from B95-8/fLuc infection model after pembrolizumab treatment confirmed co-localization of neoplastic cells (CD20^+^, white; CD30^+^, magenta) and T lymphocytes (CD8^+^ yellow; CD4^+^, green). Lymphocytes were actively proliferating (Ki67^+^, red), particularly CD8 (blue arrows). DAPI (blue) is used to mark cell nucleus. Bars correspond to 200 µm (full image) and 50 µm (inserts).

Brains explanted of ICI-treated mice showed detectable BLI signals, but this was not the case for brains of control mice (**Figures 3A, B**). Histopathology confirmed presence of EBER^+^ cells in brains of infected mice. In two cases of the ICI-treated group, both from the B95-8 model, three distinctive patterns of local CNS manifestations of EBER^+^ LPD were observed with blast-like morphological appearance (**Figures 3C, D**): (i) Intra- or perivascular spread: single tumor cells were found within the lumen of small, mostly capillary blood vessels (**Figure 3C**, blue and white inserts, brown arrows) and close to the vessel walls in small veins (**Figure 3D**, blue inserts, brown arrows), resembling patterns observed in brain manifestations of human PTLD (**Figure 3E**, Case 01 and 02**)**; (ii) Meningeal spread: coherent sheets of tumor cells forming a meningeal infiltration at the brain surface (**Figures 3C, D**) and resembling neoplastic meningitis in corresponding human diseases, e.g., EBV^+^ PTLD (**Figure 3E**, Case 03); (iii) Diffuse brain infiltration: diffusely infiltrating tumor cells formed a partially perivascular tumor mass, mixed with pre-existing brain tissue (**Figure 3C**, red insert), corresponding to the characteristic patterns of human (primary) CNS lymphoma and/or monomorphic PTLD (**Figure 3E**, Case 01 and 03). In addition to these patterns, there was also diffuse involvement of the choroid plexus in treated mice. In contrast, the control animals showed only occasionally single EBER^+^ cells associated with blood vessels but no meningeal or intraparenchymal involvement and no positive cells in the choroid plexus (**Figure 3F**). The morphological phenotype of the EBER^+^ tumor cells corresponded to the extracerebral manifestations (**Figures 3G–J**, top panels), as well as the immune phenotype with inconsistent CD30 expression, CD20 positivity, and high Ki67^+^ proliferative activity (**Figures 3G–J**, middle panels). EBNA2 and LMP1 staining indicated latently-infected EBV^+^ cell population (**Figures 3G–J** lower panels). In-depth analysis of the tumor spread to the CNS by multiplex-immunohistochemistry confirmed the B cell lineage of the blast-like tumor cells, the high proliferative activity, the partial expression of CD30, and a scarce bystanding infiltration with some CD4^+^ and CD8^+^ lymphocytes, the latter frequently co-expressing the proliferation marker Ki67 (**Figure 3K**).

### PD-1 Blockade Results in Lower Frequencies of Circulating Cytotoxic T Lymphocytes and Th Cells and Reduced Detection of PD-1 on T Cells

**Figure 4:**
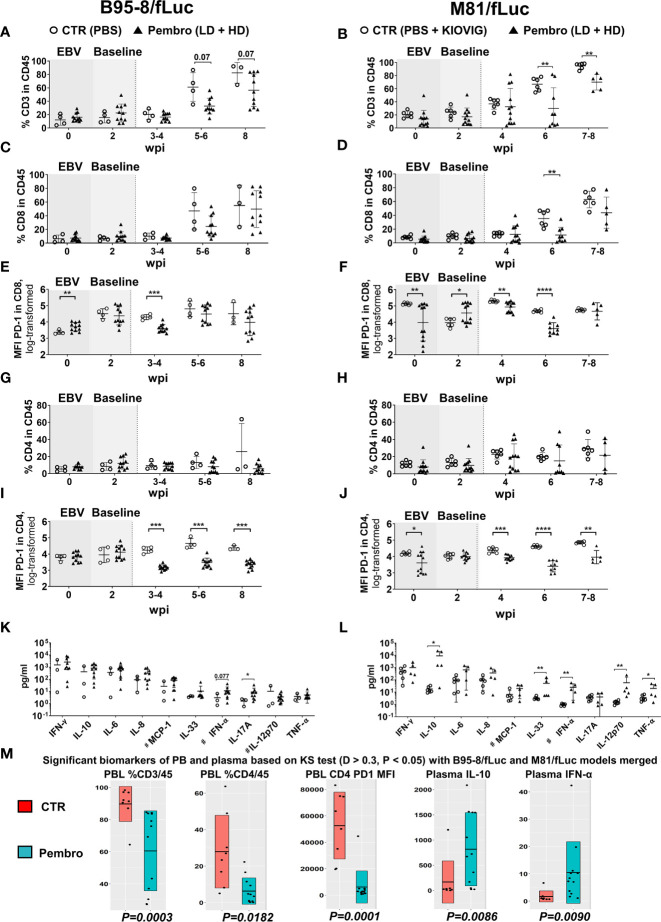
Reduced frequencies of human T lymphocytes in peripheral blood and increased concentrations of human cytokines in plasma after PD-1 blockade during active EBV infections. Peripheral blood was collected on the day prior to EBV infection (time-point 0) and 2 weeks post-infections (wpi), as baseline values for randomization (shaded graphs). Non-treated control mice (CTR, open circles) and pembrolizumab treated cohorts are shown (filled triangles; low-dose and high-dose treatments were merged for the analyses, each dot represents a mouse analyzed at the corresponding time point). After initiation of pembrolizumab treatment, peripheral blood was collected at 3–4, 5–6, and 8 wpi. The different EBV infection models (B95-8/fLuc; left panels and M81/fLuc; right panels) were analyzed separately regarding lymphocytes and cytokines and then the data was merged for power analyses and identification of markers associated with response. **(A–J)** Flow cytometry analyses of peripheral blood lymphocytes. **(A, B)** Frequencies (%) of human CD45^+^/CD3^+^ lymphocytes were reduced after pembrolizumab treatment. **(C, D)** Frequencies (%) of human CD45^+^/CD8^+^ lymphocytes were reduced after pembrolizumab treatment. **(E, F)** Mean fluorescence intensities (MFI) of PD-1 detected on the surface of CD8^+^ T cells were reduced after pembrolizumab treatment. **(G, H)** Frequencies (%) of human CD45^+^/CD4^+^ lymphocytes were reduced after pembrolizumab treatment. **(I, J)** MFI of PD-1 detected on the surface of CD4^+^ T cells were significantly reduced after pembrolizumab treatment. Statistical analyses for flow cytometry data was performed using unpaired t test with Welch’s correction. Standard deviation is indicated. *P < 0.05, **P < 0.01, ***P < 0.001, ****P < 0.0001. **(K, L)** Concentration human cytokines (pg/ml) at 8 wpi showing elevation for several cytokines in plasma of mice infected B95-8/fLuc **(K)** or M81/fLuc **(L)** treated with pembrolizumab. Mann-Whitney t test was used to compare the control and treatment groups. Standard deviation is indicated. *P < 0.05, **P < 0.01. # indicates parameters with some values being beyond the lower limit of detection (LOD), LOD was used for these samples to analyze the differences. **(M)** Global analysis for power analyses and identification of T cell and cytokine patterns at 8 wpi. The datasets from the B95-8/fLuc and M81/fLuc infection models were merged. Kolmogorov-Smirnov-Test (KS test; *D > 0.3, P < 0.05*) is depicted for flow cytometry and cytokine datasets showing markers with significant difference between control (CTR, red) and pembrolizumab treatment (Pembro, blue). P-values determined for each biomarker are indicated below the box plots. The top biomarkers associated with pembrolizumab treatment were: Reduced %CD3/CD45 (*P = 0.0003*), reduced %CD4/CD45 (*P = 0.0182*), reduced MFI of PD-1 expression on CD4^+^ cells (*P = 0.0001*), increased IL-10 (*P = 0.0086*) and IFN-α (*P = 0.0090*) levels in plasma.

Longitudinal analyses of blood samples were performed to monitor the frequencies of circulating human T cells (see **Figure S1A** for the flow cytometry gating strategy). The frequencies of huCD45^+^ hematopoietic cells at the time of EBV infections were comparable for both models (B95-8/fLuc: mean 26.4%; M81/fLuc: mean 33.7%). Relative to controls, the frequency of blood huCD45^+^ cells was significantly reduced for mice infected with M81/fLuc and treated with ICI (M81 model 7–8 wpi: CTR *vs* Pembro, *P = 0.0017*; **Figures S2A, B**; see **Tables S4**, **5** for descriptive data). Consistent with our previous observations after EBV/B95-8 infection (24), significant increase in CD3^+^ frequencies in blood (**Figures 4A, B**, **Tables S4**, **5**) and significant decrease in frequencies of CD19^+^ B cells were observed from 4 to 8 wpi (**Figures S2C, D**, **Tables S4**, **5**). ICI treated mice at 6–8 wpi, however, showed lower frequencies of CD3^+^, CD8^+^, and CD4^+^ T cells compared with controls (**Figures 4A–D, G, H**; **Tables S4**, **5**). We have observed that T cells pre-treated with pembrolizumab for 30 min *in vitro*, washed and then stained with the fluorochrome-labeled monoclonal anti-PD-1 antibody (clone EH12.2H7) showed a drastic reduction on the detectable PD-1 (**Figure S2E**). The most likely explanation is that the epitope is bound by pembrolizumab and this blocks the antibody used for immune staining of T cells, resulting in epitope “masking.” This “masking” effect was confirmed by measurement of the mean fluorescence intensity (MFI) of PD-1 analyses of T cells from 3 to 4 wpi, *i.e.* 2 weeks after initiation of therapy (**Figures 4E, F, I, J**; **Figure S1A**; **Table 2**, **Tables S4**, **5**). For both models, this PD-1 masking effect was consistently more pronounced for CD4^+^ (CTR *vs* Pembro, *P < 0.01* to *P < 0.0001*) compared to CD8^+^ T cells.

### PD-1 Blockade Results in Increased Levels of Several Human Cytokines in Plasma of Epstein–Barr Virus Infected Mice

Plasma was collected at the endpoint for analyses of human cytokines in the circulation. The levels of both pro-inflammatory (IL-6, IL-8, IFN-α, IFN-γ, MCP-1, and TNF-α) and immune-suppressive (IL-10) cytokines were elevated in ICI-treated mice (**Figures 4K, L**, **Figures S2F, G**, **Table 2**, **Table S6**). Treatment of EBV-M81/fLuc-infected mice with KIOVIG did not affect the immune reconstitution profile nor cytokine levels and, therefore, these immune-modulations were only seen in mice after EBV infection and PD-1 blockade (**Figures S2H–J**). Administration of pembrolizumab alone, in absence of EBV did not have a comparable effect with either treated or untreated groups (**Figures S2H–J**).

### Biomarkers Identified in Blood and Plasma After Immune Checkpoint Inhibition Treatment

The datasets of both EBV-models were merged for the identification of concordant biomarkers defining a response to pembrolizumab treatment (**Figure 4M**). Overall, in comparison with control mice, ICI-treated mice showed significant: (i) lower frequencies of CD3^+^/CD45^+^ and CD4^+^/CD45^+^ T cells, (ii) lower MFI of PD-1 detectable on CD4^+^ cells; (iii) elevated levels of IL-10 and IFN-α. In summary, PD-1 blockade applied during an active EBV infection resulted in lower detection of PD-1^high^ circulating lymphocytes (particularly for CD4^+^ T cells) and in a profound modulation of the cytokine levels in plasma.

### PD-1 Blockade Promotes a Quantitative Loss of CD4^+^ T Cells in Lymphatic Tissues

**Figure 5:**
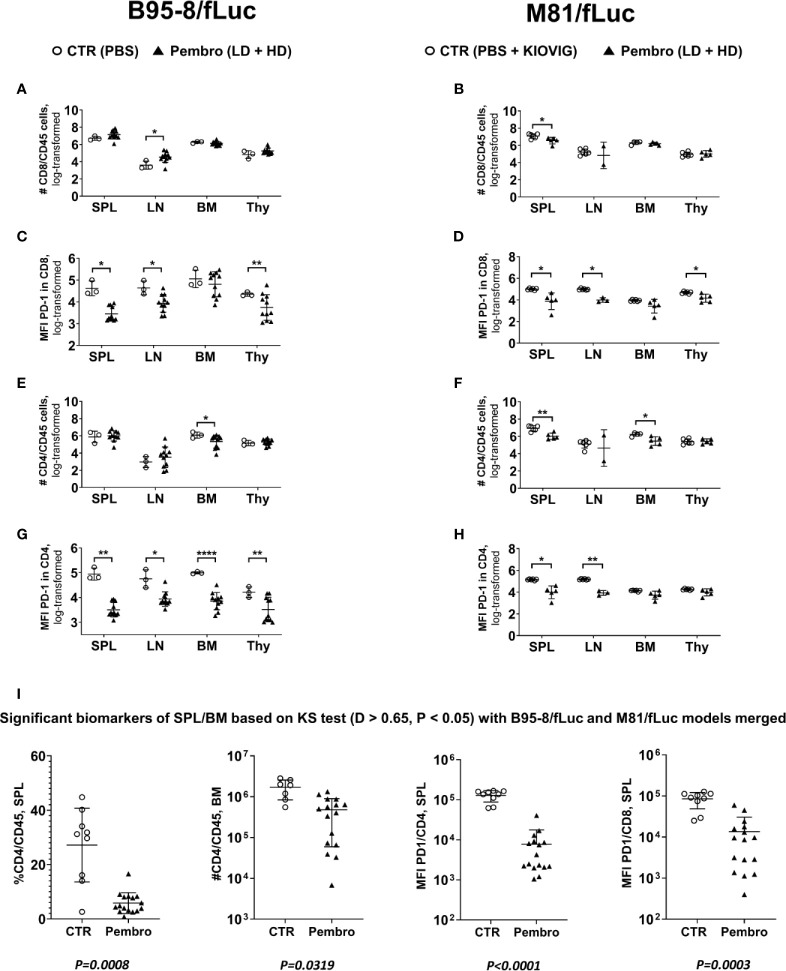
The absolute numbers of human T lymphocytes in lymphatic tissues increased for the B95-8/fLuc model and decreased for the M81/fLuc model after PD-1 blockade. The absolute numbers of viable lymphocytes recovered from spleen (SPL), lymph nodes (LN), bone marrow (BM), and thymus (Thy) were counted and, after flow cytometry analyses, the absolute numbers of each lineage was calculated. Non-treated control mice (CTR, open circles) and pembrolizumab-treated cohorts are shown (filled triangles; low-dose and high-dose treatments were merged for the analyses; each dot represents a mouse analyzed at the terminal time point). Only mice surviving until the terminal analyses at 8 wpi were included in the terminal flow cytometry analyses. **(A, B, E, F)**. Quantified total lymphocyte numbers (# cells) calculated for CD8^+^ T cells **(A, B)** and CD4^+^ T cells **(E, F)** for each organ. Note the increased numbers of lymphocytes for the B95-8/fLuc models and the decreased numbers for the M81/fLuc models for the pembrolizumab-treated mice. **(C, D, G, H)** Mean fluorescence intensity (MFI) for PD-1 detection for CD8^+^ T cells **(C, D)** and CD4^+^ T cells **(G, H)** showing overall significantly reduced PD-1 detection after pembrolizumab treatment for both models. Unpaired t test with Welch’s correction was used to compare treatment groups. *P < 0.05, **P < 0.01, ****P < 0.0001. Standard deviation is indicated. **(I)** Global power analyses and biomarker identification. The flow cytometry datasets of spleen of B95-8/fLuc and M81/fLuc models were merged. Kolmogorov-Smirnov Test (KS test; D > 0.65, *P < 0.05*) is depicted. The biomarkers identified with significant differences between control (CTR, open circles) and pembrolizumab treatment (Pembro, filled triangles) were: Lower SPL % CD4/CD45 (*P = 0.0008*); lower BM #CD4/CD45 (*P = 0.0319*); lower MFI for PD-1 in CD4^+^ cells (*P < 0.0001*); lower MFI for PD-1 in CD8^+^ cells (*P = 0.0003*). P-values determined for each marker are indicated below the graphs.

The immune composition of total human T cell counts in lymphatic tissues (SPL, LN, BM, and Thy) was performed to characterize the immunologic effects of ICI on CD8^+^ and CD4^+^ T cells in further detail (**Figure 5**, see **Tables S7**, **8** for descriptive data). For the B95-8/fLuc model, ICI treatment promoted a modest expansion of the CD8^+^ T cell compartment in lymphatic tissues (**Figure 5A**), whereas for the M81/fLuc model a significant reduction was seen (**Figure 5B**, *P < 0.05*). The CD8^+^ T cells from ICI-treated mice were PD-1^Low^, recapitulating the epitope masking effect (**Figures 5C, D**). Numbers of CD4^+^ T cells were reduced compared to control in BM of ICI-treated mice for both EBV models (*P < 0.05*), as well as in the SPL of M81 model (*P < 0.01*) (**Figures 5E, F**). For the mice surviving until the endpoint analyses, PD-1 masking of CD4^+^ T cells was more pronounced in lymphatic tissues of mice infected with B95-8 than infected with M81 (*P < 0.05 to P < 0.0001*, **Figures 5G, H**; **Tables S7**, **8**). Overall, the MFIs measured for PD-1 in CD4^+^ T cells were significantly reduced in ICI-treated mice compared to controls, although this varied for different tissues (*P < 0.05 to P < 0.0001*, **Figures 5G, H**; **Tables S7**, **8**).

### Biomarkers Identified in Lymphatic Tissues After Immune Checkpoint Inhibition Treatment

The datasets of tissue analyses of both EBV-models were merged for identification of biomarkers of response to pembrolizumab, showing: (i) lower frequencies and lower absolute numbers of CD4^+^/CD45^+^ in SPL (*P = 0.0008*) and BM (*P = 0.0319*) and (ii) lower MFI of PD-1 detection on CD4^+^ cells (*P < 0.0001*) and CD8^+^ T cells (*P = 0.0003*) (**Figure 5I**). In summary, pembrolizumab treatment after EBV infection consistently lowered the numbers of PD-1^+^CD4^+^ T cells in lymphatic tissues.

### PD-1 Blockade Promotes T Cell Exhaustion and High Frequencies of Regulatory T Cells in Spleen

**Figure 6:**
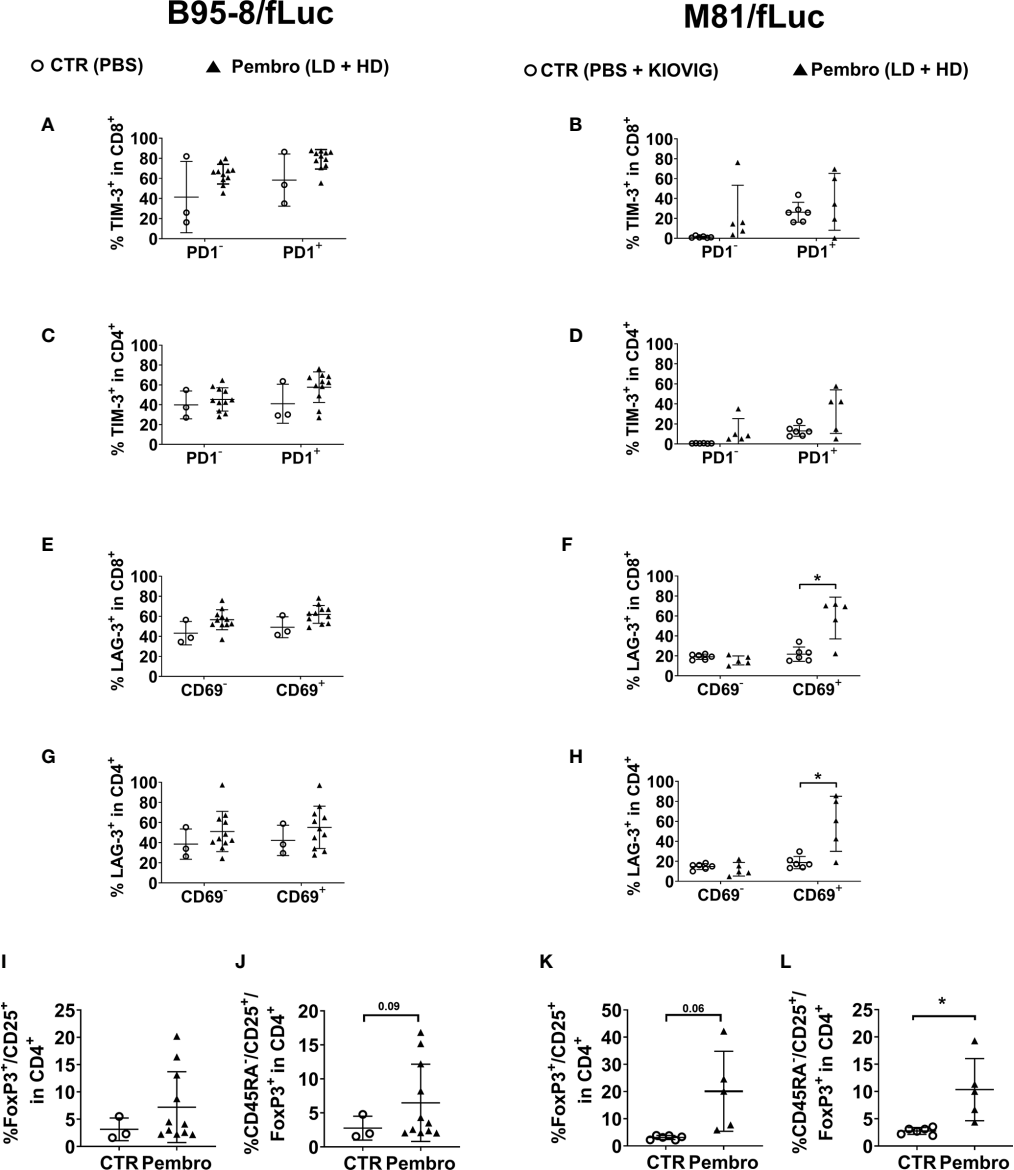
The frequencies of T cells expressing TIM-3 or LAG-3 exhaustion markers or a regulatory T cell immunophenotype increased after pembrolizumab treatment. Frequencies of marker-positive lymphocytes recovered from spleen (SPL) comparing non-treated control mice (CTR, open circles) and pembrolizumab-treated cohorts are shown (filled triangles; low-dose and high-dose treatments were merged for the analyses). Only mice surviving until the terminal analyses at 8 wpi were included in the terminal flow cytometry analyses. **(A–D)** Frequencies (%) of TIM-3^+^ in CD45^+^/CD8^+^ **(A, B)** and of TIM-3^+^ in CD45^+^/CD4^+^ **(C, D)** T cells are shown. PD-1^+^ (left side) or PD-1^−^ (right side) CD8 or CD4 subpopulations were analyzed separately. Higher proportion of TIM-3^+^ cells was observed more clearly for PD1^+^ T cells for both models. **(E–H)** Frequencies (%) of LAG-3^+^ in CD45^+^/CD8^+^ **(E, F)** and LAG-3^+^ in CD45^+^/CD4^+^ **(G, H)** T cells are shown. CD69^+^ (left side) or CD69^-^ (right side) CD8 or CD4 subpopulations were analyzed separately. Significantly higher proportion of LAG-3^+^ T cells were observed for activated CD69^+^ T cells for mice infected with M81/EBV/fLuc and treated with pembrolizumab. **(I–L)** Frequencies (%) of CD25^+^/FoxP3^+^/CD45^+^ regulatory **(I, K)** and CD45RA^-^/CD25^+^/FoxP3^+^/CD45^+^ activated regulatory T cells **(J, L)** detected within the human CD4^+^ cells for B95-8 **(I, J)** and M81 model **(K, L)**. Pembrolizumab treatment was associated with higher proportion of regulatory T cells for both models. Unpaired Welch’s t test was used to compare treatment groups. *P < 0.05. Standard deviation is indicated.

Splenocytes were analyzed to identify markers of immune exhaustion. TIM-3 analyses were included because TIM-3 cooperates with PD-1 in T cell dysfunction in chronic viral infections and PD-1^+^/TIM-3^+^ CD8^+^ T cells express high levels of IL-10 (37). For both CD8^+^ and CD4^+^ T cell sub-populations, treated mice showed higher frequencies of TIM-3^+^ cells (**Figures 6A–D**; **Tables S7**, **8**; see the gating strategy in **Figure S1B**). Higher frequencies of cells expressing TIM-3 were observed for PD-1^high^ cells, indicating that T cells evading or not masked with pembrolizumab were more exhausted. In another panel, LAG-3 was analyzed in conjunction with the activation marker CD69 (**Figures 6E–H**; **Tables S7**, **8**, see gating strategy in **Figure S1C**). LAG-3 is another checkpoint receptor that defines a potent regulatory T cell subset that occurs more frequently in cancer patients and is expanded at tumor sites (38). The frequencies of CD8^+^ and CD4^+^ T cells positive for both CD69 and LAG-3 were moderately increased for treated mice infected with B95-8/fLuc, while significantly increased for M81/fLuc infected and ICI-treated mice (**Figures 6E–H**). Altogether, these results indicated that the PD-1 blockade after EBV infection magnified T cell exhaustion. As complementary analyses, the presence of CD25^+^/FoxP3^+^/CD4^+^ regulatory T cells (Tregs) in spleen was determined. A modest increase of Tregs proportion in CD4 cells was observed for the B95-8/fLuc-infected treated mice relative to controls (**Figures 6I, J**, **Tables S7**), while the Tregs were significantly amplified in mice infected with M81/fLuc after ICI treatment (**Figures 6K, L**, **Table S8**, for gating strategy see **Figure S1D**). Therefore, both exhaustion of CD4^+^ and CD8^+^ T cells and higher frequencies of Tregs were promoted by PD-1 blockade after EBV-infection, and this signature was even more noticeable for the M81/fLuc lytic model.

### PD-1 Blockade Is Associated With Increased Densities of CD4^+^/FoxP3^+^ T Cells Within Tumors

**Figure 7:**
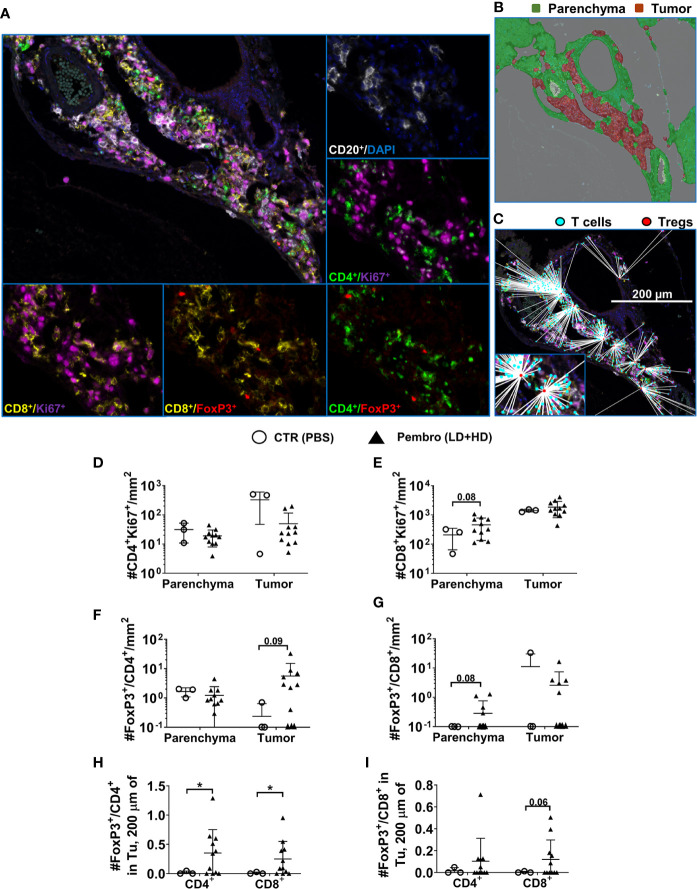
Lower overall infiltration of CD4^+^ T cells but higher density and chance of intercellular interactions of regulatory T cells in tumors of mice infected with EBV/B95-8/fLuc and treated with pembrolizumab. **(A)** Representative example of a multiplexed immunohistochemical (mIHC) staining of liver tissue of a B95-8/fLuc-infected and pembrolizumab-treated humanized mouse followed by multispectral image (MSI) analysis/color deconvolution into six channels representing CD20^+^ (white), Ki67^+^ (magenta), CD4^+^ (green), CD8^+^ (yellow), FoxP3^+^ (red), and DAPI (blue) markers. **(B)** Tissue segmentation between the perivascular tumor mass (red) and the non-neoplastic parenchyma (green). Expectedly, the tumor area corresponds with high density of proliferating CD20^+^ blast-like cells **(A, B)**. **(C)** Comprehensive distance mapping between FoxP3^+^ (red dots) and FoxP3^-^ T-cells (cyan dots). **(D)** The tumor areas of treated animals (black triangles, n = 11) showed fewer CD4^+^ T cells than controls (open circles, n = 3). **(E)** The parenchyma area is characterized by a prominent population of proliferating CD8^+^ cells, with a trend towards higher densities in treated animals (not significant). **(F)** Densities of proliferating Ki67^+^CD4^+^ Treg cells in the parenchyma or in the tumor tissue, with an increase of tumor-infiltrating FoxP3^+^/CD4^+^ Treg cells in the pembrolizumab-treated group (not significant), **(G)** that was also observed for parenchyma FoxP3^+^/CD8^+^ Treg cells (not significant). **(H)** Distance metrics between FoxP3^+^/CD4^+^ Tregs or **(I)** FoxP3^+^/CD8^+^ Tregs revealing significantly increased average number of FoxP3^+^/CD4^+^ within a radius of 200 µm around any single CD4^+^ or CD8^+^ T cells residing in the tumor area for pembrolizumab-treated (black triangles) mice compared with control (open circles) mice. Unpaired t test with Welch’s correction was applied to calculate statistical significance. Standard deviation is indicated. *P < 0.05.

The tumor immune microenvironment was assessed using a multiplexed immunohistochemistry approach. The cell densities and distance metrics within the tumor or parenchyma areas using tissues obtained from B95-8/fLuc-infected mice were analyzed (**Figures 7A–C**, representative example of immune cell phenotyping in a liver tumor and adjacent parenchyma by co-staining for DAPI, CD20, CD8, CD4, the proliferation marker Ki67, and the Treg marker FoxP3; for corresponding analyses in spleen tissue see **Figure S3A**). The density of proliferating CD4^+^ T cells showed an overall modest reduction for ICI-treated mice compared with controls, however, this did not occur for CD8^+^ T cells (**Figures 7D, E**, liver; **Figures S3C, D**, spleen; **Table S9**). Further, higher densities of CD4^+^/FoxP3^+^ T cells, were observed within the tumor area (**Figure 7F**, liver; **Figure S3E**, spleen). CD8^+^/FoxP3^+^ T cells, another subset of immunosuppressive cells, were also present in liver parenchyma of ICI-treated, but not in the control group (**Figure 7G**, liver; **Figure S3F**, spleen). The analysis of distance metrics between different T cell subtypes in liver tissues (**Figure 7C**, liver; for spleen see **Figure S3B**) showed that the average presence of CD4^+^/FoxP3^+^ Tregs in up to 200 µm radius around any single FoxP3^-^ T cell (both CD4^+^/FoxP3^−^ or CD8^+^/FoxP3^−^) was higher in the pembrolizumab-treated group than in controls (*P < 0.05*, **Figures 7H, I**, liver; **Table 2**, **Table S9**), suggesting that immune regulatory T cells with CD4^+^/FoxP3^+^ and CD8^+^/FoxP3^+^ phenotypes were enriched. This was observed in the immediate microenvironment of T-effector and helper cells of ICI-treated mice with more advanced tumors indicating a higher chance of direct cell-to-cell interactions, potentially associated with immune suppression and aggravation of EBV spread upon PD-1 blockade.

## Discussion

Several human malignancies are initiated by viral oncogenesis, such as EBV, human papillomavirus (HPV), hepatitis B virus (HBV), and hepatitis C virus (HCV). Currently, there are several ongoing clinical trials of ICIs to combat virus-associated cancers (39). For example, pembrolizumab is currently being tested for the treatment of EBV^+^ nasopharyngeal carcinoma (NPC), EBV^+^ Non-Hodgkin’s disease, EBV^+^ NK/T cell lymphoma, EBV^+^ gastric cancer, HPV^+^ cervical cancer, and HPV^+^ head and neck squamous cell carcinoma (39). One potential risk to be evaluated is that viral immune evasion mechanisms may develop to counteract the human immune responses affecting the mode of action of ICI. On the other hand, a substantial amount of cancer patients treated with ICI occasionally show HPD, but the etiological reasons (such as for example action of underlying infections) are not yet fully understood (40–42). To avoid HPD or to mitigate its risks, the causes and early predictors associated with this phenomenon require investigation. The tumor burden, level of immune infiltration in the tumor, quality of immune responses, immune biomarkers, and existence immunomodulatory PD-1 positive lymphocytes have all been associated with HPD (43–47). Wong et al. reported a case series of four patients with advanced hepatocellular carcinoma with chronic HBV infections treated with ICIs and demonstrating rapid radiological progression and HPD (48). A recent review summarizing the results on the safety and efficacy of ICI in HBV/HCV-infected advanced cancer patients concluded that it was safe and effective, but warned about the reactivation of hepatitis virus in some cases (49). Currently, there is no clear relationship embracing ICI, cancer, chronic infections, irAEs, and HPD. Recently, it was shown in a mouse model and in patients with tuberculosis that PD-1 blockade mounted rampant CD4^+^ T helper type 1 (Th1) responses driving lethal disseminated tuberculosis (50). In another alarming report, metastatic melanoma patient suffered fatal encephalitis after PD-1 blockade therapy with pembrolizumab. The affected brain tissue contained lymphocytes infected with EBV with a concurrent infiltration of memory cytotoxic CD4^+^ and CD8^+^ T cells reactive against EBV (51). Therefore, as these clinical case reports are summing up, there is a significant need to examine pre-clinically the potency and potential side effects of ICIs affecting virus-related malignancies.

There were previously two discordant reports in the literature concerning the effects of PD-1 blockade tested pre-clinically in mouse models for the treatment of EBV-related PTLD and DLBCL (**Table 1**). Corroborating our results, Münz and colleagues showed that fully humanized mice infected with the B95-8 strain and treated with a PD-1 blocking mAb developed rampant EBV spread and inflammatory reactions (25) (**Table 1**). Here, we showed that these results could be reproduced with pembrolizumab and for both the B95-8 (preferentially latent) and M81 (preferentially lytic) EBV infection models. Therefore, we suggest that PD-1 blocking in the setting of a primary infection and T cell priming can lead to uncontrolled and widespread EBV infections and development of neoplasias (PTLD and DLBCL-like tumors). Incidentally, pembrolizumab treatment affected even more drastically mice infected with M81, resulting in significantly accelerated and increased death rates. One aspect that has to be acknowledged in respect to the fully humanized mouse models of EBV infection presented by Chatterjee et al. (25) and by this current work is that their immune reconstitution at the time of infection was biased towards a high abundance of B cells (>50%) relative to T cells (<30%). These high levels of B cells, which are not seen in humans after transplantation, may have predisposed a more aggressive PTLD development in the mice. Taking this bias in consideration, we may be overestimating the substantial detrimental immunomodulatory effects caused by pembrolizumab.

Of note, there is a clear discrepancy between our results and those of Kenney and collaborators who observed lower tumor burden after PD-1 blockade when using the M81 viral strain in a not fully humanized model (27) (**Table 1**). The most straightforward explanation for the different results is that they pre-treated the mature B and T cell mixture *in vitro* with the PD-1 and CTLA-4 blocking mAbs and then administered the cells into the mice. Therefore, this *ex vivo* pre-treatment *per se* may have stimulated the adoptively transferred human T cells to become more xeno-reactive in the mice and to expand more strongly than the co-administered infected B cells. The activated and expanded T cells could eventually kill or inhibit the outgrowth of EBV-infected malignant B cells *in vivo*. This xenograft model was carried on only for 4 weeks, as the adoptively transferred human T cells can strongly react against mouse tissues and eventually result into lethal xeno-GvHD. This adoptive transfer mouse model strongly deviates from PTLD in humans, since after HCT the adoptively transferred T cells from the donor are at least partially matched through the major histocompatibility complexes (MHCs) to the recipient and there is also donor-derived thymopoiesis.

On the other hand, in fully humanized mouse models reported by Chatterjee and by this current work, the human T cells undergo an endogenous development (18, 25). The hematopoietic progenitors home in the bone marrow, travel through the thymus for positive and negative selection of functional non self-reactive T cell receptors (TCRs). TCR restriction is mediated by the MHC of the mouse and of the HSC donor. The developed human TCR^+^/CD4^+^ T cells and in lower frequencies TCR^+^/CD8^+^ T cells, eventually home in the lymphatic tissues and organs. Upon vaccination of fully humanized mice, the naïve human T cells show typical signs of priming, and change their immunophenotype from naïve to memory subtypes (29). Functional human B cells binding to viral antigens also develop in humanized mice with full maturation after class switch (30). Thus, these HIS models reflect homeostatic modulations and are multidimensional, as T cells with diverse functions and at several stages of differentiation co-exist in different tissues. From the preclinical perspective, it is indeed more challenging to perform studies in fully humanized mice, but these complexities reflect intrinsic mechanisms that may be missed in *in vitro* or in simpler xenograft models.

Primarily, the main mechanistic prospect of ICI is the release of inhibited CD8^+^ memory TILs through the blockade of the PD-1/PD-L1 axis in order to revert their functionality (3, 43). Nonetheless, less is reported about the blockade of the PD-1/PD-L1 axis during the priming and expansion phase of naïve CD8^+^ T cells, which occurs in humans and mice with acute infections, such as lytic EBV infections (24, 25). Indeed, it has previously been shown that downregulation of PD-1 expression prevented T cell proliferation by accelerating a T cell early differentiation (52).

The significant amplification of several human cytokines that we observed in the plasma of M81-infected mice after PD-1 blockade (IL-10, IL-33, IFN-α, IL-12p70, and IL-1β) revealed the ignition of a profound inflammatory response. The spread of viral infection systemically and the remarkable infiltration of EBV^+^ PTLD into the CNS indicated a defaulted TIL response. This worrisome finding suggested that the aggravated infection and/or inflammation could potentially weaken the blood-brain-barrier. Of note, we observed that LAG-3 was strongly upregulated on CD8^+^ and CD4^+^ T cells after PD-1 blockade. Therefore, it is possible that the viral immune escape maneuvered the T cell exhaustion through upregulation of other suppressive receptors, like LAG-3.

In addition, our data also underscored bystander effects involving the participation of CD4^+^PD-1^+^ T cells. Their collapse and/or conversion into dysfunctional exhausted cells or Tregs likely contributed to viral escape and to irAEs. The role of Tregs seemed to be particularly critical within the tumor microenvironment on the initial stages of tumor development. Although we have not yet identified an EBV-specific molecular and immunologic mechanism by which PD-1 blockade may have affected the CD4^+^ Tregs function, some correlations exist with neoplasias caused by EBV. For example, *in vitro* studies showed that EBV latency III–transformed B cells promoted expansion of autologous FoxP3^+^ CD39^high^ PD-1^+^ CTLA-4^+^ Helios^+^ GITR^+^ LAG-3^+^ CD4 Tregs (53). Another study showed that homing of Tregs in EBV^+^ HL tumor microenvironment is significantly increased around EBV-infected cell nests (54). Tregs also effectively populate EBV^+^ gastric carcinoma tumors and express PD-1 (55). In EBV^+^ NPC, EBNA-1-and LMP2-specific CD8^+^ CTL responses can be suppressed *in vitro* by Tregs and their depletion with a recombinant fusion protein (Ontak) reverted this immune suppression (56). Therefore, Treg depletion combined with PD-1 blockade could be eventually tested, with the caveat that persistent CD25 immune depletion can lead to immune deficiency. Future studies are needed to clarify the participation of Tregs in EBV^+^ HPD.

Remarkably, we also noticed a conspicuous presence of CD8^+^/FoxP3^+^ cells in tumors, endorsing that TILs can be subverted to immunosuppressive Tregs. Incidentally, CD25^+^CD8^+^FoxP3^+^ Tregs were previously described as cells showing rapid expansion in blood and tissues of rhesus macaques during the acute phase of simian immune deficiency (SIV) infection (57). CD8^+^ Tregs were also observed in humans infected with the human immune deficiency virus and expressed low levels of granzyme B and perforin, suggesting that they did not possess killing potential but were related to immune suppressor functions (57). Thus, development of virus-induced CD8^+^ Tregs during acute infection may be a mechanism to curb the primary immune response so that the pathogen can take hold. Nonetheless, variability of PD-1^+^ T cell populations emerging in different infections has been shown, pointing to diverse mechanisms inducing suppressive T cell types. For example, for acute influenza A virus infection, mainly central memory CD8^+^ T cells with a PD-1^+^2B4^+^CXCR5^+^ phenotype thrive after viral clearance (58). In contrast, CD8^+^ T cells with these characteristics were completely absent in viremic HIV infected individuals (58).

Concluding, our study endorsed preclinical (25) and clinical observations (51) regarding the interaction of EBV infection with PD-1 blockade in sparking EBV spread. One novel mechanistic insight provided is the possible role of immunosuppressive PD1^+^CD4^+^ Tregs cells. One limitation of our current studies was that a single agent, pembrolizumab, was tested for ICI and in a single treatment schedule. It is possible that other ICIs or treatments at different doses or time points may give different results. For instance, blocking PD-L1 on the tumor side may produce less immunosuppressive effects on T cell effects primed against EBV^+^ PTLD or DLBCL. Additional studies in humanized mice using ICI drugs in the preclinical testing pipeline can provide predictive information for very critical decisions that clinicians will eventually have to make, i.e., to opt for single or combinations of targets for treatment of EBV^+^ tumors. From the translational perspective, as clinical trials against different malignancies exploring the use of ICI in combination with chemotherapy, rituximab, or small drugs against EBV^+^ malignancies are emerging, it would be recommendable to first evaluate if the patients have active EBV infections and second to monitor the patients for possible EBV reactivations. Therefore, these findings warrant careful preclinical assessment of the EBV status in patients in order to minimize the side effects and improve the outcomes for patients treated with PD-1 blockade to avoid irAEs and HPD. Noteworthy, this report illustrates discordant interpretations of preclinical results (**Table 1**), advocating for a transparent reporting of the methodological approaches used towards the “Minimal Information for Standardization of Humanized Mice” (MISHUM) so that the field can further evolve (18).

## Data Availability Statement

The original contributions with the descriptive data and corresponding statistical analyses presented in the study are included in the article/**Supplementary Material**; further inquiries can be directed to the corresponding author.

## Ethics Statement

Collection of CB was performed at the Department of Gynecology and Obstetrics (Hannover Medical School) and obtained according to study protocols approved by the Ethics Committee of the Hannover Medical School and after informed consent obtained from the mothers (Study protocol Nr.4837). The patients/participants provided their written informed consent to participate in this study. All experiments involving mice were approved by the Lower Saxony Office for Consumer Protection and Food Safety—LAVES (permit number: 16/2222) and performed in accordance with the German animal welfare act and the EU-directive 2010/63.

## Author Contributions

VV, ST, and SD conducted the experiments, analyzed the data, and wrote the first manuscript draft and revised the manuscript. SK and MM-H performed the computational analysis. MK, AS, and JB-W conducted the experiments and helped with humanized mouse work. NK performed the histological analysis. YD and CM performed the EBV histological analysis. BE-V and AD conducted the cytokine analysis. CK provided the CB units. SL and AB assisted with the mouse breeding and *in vivo* optical imaging analyses. JK provided the concept for the dosage of pembrolizumab for use in humanized mice. FK conducted the statistical analysis. WH provided the EBV/B95-8/fLuc EBV strain and technical assistance. H-JD provided the EBV/M81/fLuc stain and technical assistance. RS and FF planned the project, designed the experiments, obtained funding and regulatory approvals, enrolled the collaborators, interpreted the data, and wrote, edited, and revised the manuscript. All authors contributed to the article and approved the submitted version.

## Funding

This work was financed by grants of the German Center for Infections Research (DZIF-TTU07.803 and DZIF-TTU07.805 to RS), by a research grant of the “The Jackson Laboratory” and a research grant from Else Kröner-Fresenius/Fortra (T04 to RS). MK received DZIF MD stipends. ST received a RegSci Ph.D. fellowship. FF was funded by the German Ministry of Education and Research (BMBF), DLR project management (e:Med) grants #01ZX1710A and #01ZX1608A.

## Conflict of Interest

The authors declare that the research was conducted in the absence of any commercial or financial relationships that could be construed as a potential conflict of interest.

## Supplementary Material

Supplementary Table 1 Data presented in **Figures 1B, C** regarding survival of humanized mice.

Supplementary Table 2 Data presented in **Figures 1J, L**. Descriptive statistics regarding the bioluminescence signal quantified as photons per second (p/sec).

Supplementary Table 3 Data presented in **Figures 1K, M**. Descriptive statistics regarding the RT-qPCR analyses.

Supplementary Table 4 Data presented in **Figures 4A, C, E, G, I**. Descriptive statistics regarding the B95-8 model for blood kinetic analyses of human immunophenotypic markers measured by flow cytometry.

Supplementary Table 5 Data presented in **Figures 4B, D, F, H, J**. Descriptive statistics regarding the M81 model for blood kinetic analyses of human immunophenotypic markers measured by flow cytometry.

Supplementary Table 6 Data presented in **Figures 4K, L**. Descriptive statistics regarding the analyses of human cytokines measured in plasma of mice.

Supplementary Table 7 Data presented in **Figures 5A, C, E, G** and **6A, C, E, G, I, J**. Descriptive statistics regarding the B95-8 model for tissue analyses of human immunophenotypic markers measured by flow cytometry.

Supplementary Table 8 Data presented in **Figures 5B, D, F, H** and **6B, D, F, H, K, L**. Descriptive statistics regarding the M81 model for tissue analyses of human immunophenotypic markers measured by flow cytometry.

Supplementary Table 9 Data presented in **Figures 7D–I**. Descriptive statistics regarding tissue multiplex IHC analyses of spleen and liver tissues explanted from B95-8-infected mice.

Supplementary Table 10 Antibodies used for flow cytometry and histopathology analyses.
