# Supplementary material for: PD-1 Blockade Aggravates Epstein–Barr Virus+ Post-Transplant Lymphoproliferative Disorder in Humanized Mice Resulting in Central Nervous System Involvement and CD4+ T Cell Dysregulations
Source: Front Oncol. 2021 Jan 12;10:614876. doi: 10.3389/fonc.2020.614876 (PMC7837057; doi:10.3389/fonc.2020.614876)
Supplement: Supplementary Table 1 — Data presented in Figures 1B, C regarding survival of humanized mice. [file Table_1.pdf]

Supplementary Table 1. Survival of humanized mice infected with B95-8/fLuc and M81/fLuc (survival days post-infection; 56 days was the terminal endpoint; early deaths are underlined). CTR – control group, LD – low dose, HD – high dose of Pembrolizumab.

Survival				Survival		
EBV-B95-8/fLuc				EBV-M81/fLuc		
Group	Mouse ID	Days post-infection		Group	Mouse ID	Days post-infection
**CTR (PBS)**	747	56		**CTR (PBS)**	114	56
**CTR (PBS)**	748	56		**CTR (PBS)**	122	56
**CTR (PBS)**	1750	42		**CTR (PBS)**	145	56
**CTR (PBS)**	1751	56		**CTR (KIOVIG)**	119	56
**Pembro (LD)**	626	56		**CTR (KIOVIG)**	120	56
**Pembro (LD)**	632	56		**CTR (KIOVIG)**	121	56
**Pembro (LD)**	1752	56		**Pembro (LD)**	125	56
**Pembro (LD)**	1767	56		**Pembro (LD)**	126	56
**Pembro (LD)**	1768	56		**Pembro (LD)**	144	53
**Pembro (HD)**	625	56		**Pembro (LD)**	793	47
**Pembro (HD)**	631	56		**Pembro (LD)**	794	42
**Pembro (HD)**	746	56		**Pembro (LD)**	795	53
**Pembro (HD)**	1769	56		**Pembro (HD)**	105	42
**Pembro (HD)**	1770	56		**Pembro (HD)**	106	33
**Pembro (HD)**	1771	56		**Pembro (HD)**	107	28
				**Pembro (HD)**	796	47
				**Pembro (HD)**	886	51
				**Pembro (HD)**	887	42
