# Supplementary material for: PD-1 Blockade Aggravates Epstein–Barr Virus+ Post-Transplant Lymphoproliferative Disorder in Humanized Mice Resulting in Central Nervous System Involvement and CD4+ T Cell Dysregulations
Source: Front Oncol. 2021 Jan 12;10:614876. doi: 10.3389/fonc.2020.614876 (PMC7837057; doi:10.3389/fonc.2020.614876)
Supplement: Supplementary Table 2 — Data presented in Figures 1J, L . Descriptive statistics regarding the bioluminescence signal quantified as photons per second (p/sec). [file Table_2.pdf]

Supplementary Table 2. Bioluminescence imaging analyses.

**t test with Welch's correction**						
**EBV-B95-8/fLuc - p/sec**						
**Week post-infection**	**CTR, N1**	**Pembro, N2**	**Mean 1**	**Mean 2**	**Mean Diff,**	**Adjusted P-Value**
**2**	4	11	5,785	5,991	0,205	0,2363
**4**	4	11	7,865	7,187	-0,6785	***0,0152***
**6**	4	11	7,712	8,328	0,6163	0,1116
**8**	3	11	7,354	9,322	1,968	***<0,0001***
**EBV-M81/fLuc - p/sec**						
**Week post-infection**	**CTR, N1**	**Pembro, N2**	**Mean 1**	**Mean 2**	**Mean Diff,**	**Adjusted P-Value**
**2**	6	12	7,228	5,759	-1,469	0,1055
**4**	6	12	7,596	7,527	-0,06892	0,9346
**6**	6	8	10,16	10,93	0,7739	0,2439
**7-8**	6	4	9,843	11,65	1,809	0,1387

** - original values were log-transformed before statistical tests*
