# Supplementary material for: PD-1 Blockade Aggravates Epstein–Barr Virus+ Post-Transplant Lymphoproliferative Disorder in Humanized Mice Resulting in Central Nervous System Involvement and CD4+ T Cell Dysregulations
Source: Front Oncol. 2021 Jan 12;10:614876. doi: 10.3389/fonc.2020.614876 (PMC7837057; doi:10.3389/fonc.2020.614876)
Supplement: Supplementary Table 3 — Data presented in Figures 1K, M . Descriptive statistics regarding the RT-qPCR analyses. [file Table_3.pdf]

Supplementary Table 3. RT-qPCR analyses calculated as EBV copies per µg DNA. Samples obtained for: B95-8 at end-point (8 wpi); M81 at end-point (8 wpi) or post-mortem (5, 7 wpi).

**t test with Welch's correction**						
**EBV-B95-8/fLuc, PCR, copies per mcg DNA**						
**Tissue**	**CTR N1**	**Pembro N2**	**Mean 1**	**Mean 2**	**Mean Diff,**	**Adjusted P-Value**
Spleen*	4	8	4,368	5,257	0,8889	0,1990
Bone Marrow*	3	8	3,867	4,865	0,9978	0,1110
**EBV-M81/fLuc, PCR, copies per mcg DNA**						
**Tissue**	**CTR , N1**	**Pembro , N2**	**Mean 1**	**Mean 2**	**Mean Diff,**	**Adjusted P-Value**
Spleen*	6	8	3,655	4,872	1,217	***0,0156***
Bone Marrow*	6	8	2,829	3,998	1,168	***0,0283***

** - original values were log-transformed before statistical tests*
