# Supplementary material for: PD-1 Blockade Aggravates Epstein–Barr Virus+ Post-Transplant Lymphoproliferative Disorder in Humanized Mice Resulting in Central Nervous System Involvement and CD4+ T Cell Dysregulations
Source: Front Oncol. 2021 Jan 12;10:614876. doi: 10.3389/fonc.2020.614876 (PMC7837057; doi:10.3389/fonc.2020.614876)
Supplement: Supplementary Table 4 — Data presented in Figures 4A, C, E, G, I . Descriptive statistics regarding the B95-8 model for blood kinetic analyses of human immunophenotypic markers measured by flow cytometry. [file Table_4.pdf]

**Supplementary Table 4. Kinetics of human immunophenotypic markers measured by flow cytometry analyses of blood cells for B95-8 model.** Analyses at several time points for control (N=4, 0-6 wpi; N=3, wpi 8) and treated mice (N=11).

| **B95-8** | **Phenotype** | **Mean** | **SD** | **Mean** | **SD** | **Mean Difference** | **Unpaired Welch's t test** |
|---|---|---|---|---|---|---|---|
| **Time-point, wpi** | **Markers** | **CTR** | **CTR** | **Pembro (merged)** | **Pembro (merged)** | **CTR vs Pembro (merged)** | **CTR vs Pembro (merged)** |
| **0** | %CD45 | 21,90 | 17,25 | 28,06 | 10,40 | -6,155 | 0,5407 |
| 0 | %CD3/CD45 | 12,13 | 8,04 | 16,08 | 6,31 | -3,955 | 0,4196 |
| **0** | %CD19/CD45 | 78,65 | 4,41 | 75,30 | 4,59 | 3,35 | 0,2489 |
| **0** | %CD4/CD45 | 5,27 | 2,84 | 7,90 | 2,73 | -2,632 | 0,1674 |
| **0** | %CD8/CD45 | 6,45 | 5,36 | 7,35 | 4,54 | -0,9007 | 0,7775 |
| **0** | CD4/PD1 MFI* | 3,777 | 0,1849 | 3,822 | 0,2931 | 0,045 | 0,7333 |
| **0** | CD8/PD1 MFI* | 3,370 | 0,1239 | 3,701 | 0,2666 | 0,3314 | ***0,0070*** |
| **2** | %CD45 | 23,93 | 8,00 | 27,17 | 13,13 | -3,248 | 0,5779 |
| **2** | %CD3/CD45 | 15,55 | 7,87 | 22,49 | 13,42 | -6,933 | 0,2489 |
| **2** | %CD19/CD45 | 74,35 | 6,92 | 69,25 | 15,14 | 5,095 | 0,3916 |
| **2** | %CD4/CD45 | 8,42 | 4,63 | 12,03 | 6,85 | -3,619 | 0,2765 |
| **2** | %CD8/CD45 | 6,64 | 2,89 | 9,83 | 7,15 | -3,195 | 0,2408 |
| **2** | CD4/PD1 MFI* | 3,947 | 0,4790 | 4,117 | 0,4162 | 0,1701 | 0,5583 |
| **2** | CD8/PD1 MFI* | 4,508 | 0,2992 | 4,377 | 0,5613 | -0,1310 | 0,5742 |
| **3, 4** | %CD45 | 24,50 | 8,72 | 17,00 | 7,35 | 7,521 | 0,1889 |
| **3, 4** | %CD3/CD45 | 12,13 | 7,63 | 16,41 | 5,94 | 3,741 | 0,4203 |
| **3, 4** | %CD19/CD45 | 78,33 | 9,38 | 77,72 | 7,44 | -3,193 | 0,5692 |
| **3, 4** | %CD4/CD45 | 9,37 | 4,53 | 8,63 | 3,73 | 0,7443 | 0,7813 |
| **3, 4** | %CD8/CD45 | 10,10 | 4,52 | 7,41 | 2,57 | 2,69 | 0,3277 |
| **3, 4** | CD4/PD1 MFI* | 4,245 | 0,1963 | 3,173 | 0,1584 | -1,072 | ***0,0003*** |
| **3, 4** | CD8/PD1 MFI* | 4,301 | 0,1554 | 3,606 | 0,2450 | -0,6956 | ***0,0001*** |
| **5, 6** | %CD45 | 20,60 | 14,12 | 16,48 | 6,19 | 4,151 | 0,6050 |
| **5, 6** | %CD3/CD45 | 61,20 | 21,51 | 32,76 | 11,83 | 28,44 | *0,0715* |
| **5, 6** | %CD19/CD45 | 29,08 | 23,58 | 57,52 | 13,20 | -23,56 | 0,1090 |
| **5, 6** | %CD4/CD45 | 13,05 | 7,19 | 7,93 | 7,02 | 5,111 | 0,2727 |
| **5, 6** | %CD8/CD45 | 47,08 | 26,80 | 24,36 | 15,58 | 22,72 | 0,1893 |
| **5, 6** | CD4/PD1 MFI* | 4,658 | 0,3002 | 3,467 | 0,2850 | -1,191 | ***0,0009*** |
| **5, 6** | CD8/PD1 MFI* | 4,806 | 0,4794 | 4,486 | 0,5555 | -0,3202 | 0,3143 |
| **8** | %CD45 | 30,57 | 11,79 | 40,29 | 19,74 | -9,727 | 0,3262 |
| **8** | %CD3/CD45 | 82,43 | 15,55 | 56,43 | 24,29 | 26,01 | *0,0738* |
| **8** | %CD19/CD45 | 13,75 | 13,65 | 36,01 | 19,58 | -22,26 | *0,078* |
| **8** | %CD4/CD45 | 25,64 | 33,00 | 5,54 | 5,41 | 20,11 | 0,4019 |
| **8** | %CD8/CD45 | 54,97 | 29,86 | 49,72 | 26,93 | 5,248 | 0,8011 |
| **8** | CD4/PD1 MFI* | 4,382 | 0,1414 | 3,357 | 0,2444 | -1,025 | ***0,0001*** |
| **8** | CD8/PD1 MFI* | 4,507 | 0,6777 | 3,979 | 0,8093 | -0,5288 | 0,3194 |

** - original values were log-transformed before statistical tests*
