# Supplementary material for: PD-1 Blockade Aggravates Epstein–Barr Virus+ Post-Transplant Lymphoproliferative Disorder in Humanized Mice Resulting in Central Nervous System Involvement and CD4+ T Cell Dysregulations
Source: Front Oncol. 2021 Jan 12;10:614876. doi: 10.3389/fonc.2020.614876 (PMC7837057; doi:10.3389/fonc.2020.614876)
Supplement: Supplementary Table 5 — Data presented in Figures 4B, D, F, H, J . Descriptive statistics regarding the M81 model for blood kinetic analyses of human immunophenotypic markers measured by flow cytometry. [file Table_5.pdf]

Supplementary Table 5. Kinetics of human immunophenotypic markers measured by flow cytometry analyses of blood cells for M81 model. Analyses at several time points in control (N=6) and treated mice (N=12, wpi 0-4; N=9, wpi 6; N=5, wpi 8).

**M81**	**Phenotype**	**Mean**	**SD**	**Mean**	**SD**	**Mean Difference**	**Unpaired Welch's t test**
**Time-point, wpi**	**Markers**	**CTR (PBS+ KIOVIG)**	**CTR (PBS+ KIOVIG)**	**Pembro (merged)**	**Pembro (merged)**	**CTR vs Pembro (merged)**	**CTR vs Pembro (merged)**
**0**	%CD45	34,13	14,60	34,13	14,60	-0,29	0,9575
**0**	%CD3/CD45	20,55	5,14	13,90	12,92	6,65	0,1400
**0**	%CD19/CD45	66,85	2,92	75,25	16,34	-8,41	0,1251
**0**	%CD4/CD45	11,52	3,04	7,99	8,17	3,53	0,2049
**0**	%CD8/CD45	8,45	2,21	5,55	4,54	2,91	*0,0869*
**0**	CD4/PD1 MFI*	4,186	0,089	3,608	0,656	-0,577	**0,0114**
**0**	CD8/PD1 MFI*	5,128	0,065	3,982	1,143	-1,146	***0,0052***
**2**	%CD45	31,45	22,33	31,45	22,33	-14,73	*0,0808*
**2**	%CD3/CD45	23,95	8,12	17,18	13,35	6,78	0,2023
**2**	%CD19/CD45	68,83	9,37	74,04	12,00	-5,20	0,3413
**2**	%CD4/CD45	13,24	4,55	9,87	8,17	3,37	0,2783
**2**	%CD8/CD45	9,67	3,85	6,51	5,49	3,17	0,1784
**2**	CD4/PD1 MFI*	4,039	0,161	3,994	0,220	-0,044	0,6358
**2**	CD8/PD1 MFI*	3,978	0,2580	4,564	0,5997	0,5863	***0,0107***
**4**	%CD45	29,57	22,10	29,57	22,10	-16,11	*0,0565*
**4**	%CD3/CD45	36,85	7,97	32,58	27,71	4,27	0,6288
**4**	%CD19/CD45	39,45	9,58	49,54	29,94	-10,09	0,3238
**4**	%CD4/CD45	22,63	5,41	19,26	15,50	3,38	0,5087
**4**	%CD8/CD45	12,87	2,64	12,38	12,56	0,50	0,8978
**4**	CD4/PD1 MFI*	4,371	0,159	3,921	0,167	-0,4499	***0,0002***
**4**	CD8/PD1 MFI*	5,291	0,068	4,932	0,320	-0,3595	***0,0026***
**6**	%CD45	15,82	8,87	15,82	8,87	17,13	***0,0081***
**6**	%CD3/CD45	66,87	9,99	29,90	31,68	36,96	***0,0083***
**6**	%CD19/CD45	25,33	8,46	56,06	30,22	-30,73	***0,0244***
**6**	%CD4/CD45	20,03	4,52	15,01	18,50	5,02	0,4544
**6**	%CD8/CD45	35,38	11,64	11,48	11,12	23,90	***0,0024***
**6**	CD4/PD1 MFI*	4,614	0,065	3,403	0,339	-1,212	***<0,0001***
**6**	CD8/PD1 MFI*	4,687	0,077	3,609	0,363	-1,078	***<0,0001***
**7, 8**	%CD45	15,46	8,67	15,46	8,67	44,84	***0,0017***
**7, 8**	%CD3/CD45	94,85	4,55	70,00	12,06	24,85	***0,0075***
**7, 8**	%CD19/CD45	1,47	2,53	13,44	8,74	-11,97	0,0680
**7, 8**	%CD4/CD45	29,22	10,80	21,47	17,96	7,74	0,4289
**7, 8**	%CD8/CD45	63,03	12,00	43,78	22,60	19,25	0,1386
**7, 8**	CD4/PD1 MFI*	4,842	0,079	3,960	0,405	-0,882	***0,0074***
**7, 8**	CD8/PD1 MFI*	4,752	0,069	4,663	0,528	-0,089	0,727

** - original values were log-transformed before statistical tests*
