# Supplementary material for: PD-1 Blockade Aggravates Epstein–Barr Virus+ Post-Transplant Lymphoproliferative Disorder in Humanized Mice Resulting in Central Nervous System Involvement and CD4+ T Cell Dysregulations
Source: Front Oncol. 2021 Jan 12;10:614876. doi: 10.3389/fonc.2020.614876 (PMC7837057; doi:10.3389/fonc.2020.614876)
Supplement: Supplementary Table 6 — Data presented in Figures 4K, L . Descriptive statistics regarding the analyses of human cytokines measured in plasma of mice. [file Table_6.pdf]

Supplementary Table 6. Endpoint cytokines levels. Levels were measured for B95-8/fLuc infected control (N=3) and Pembrolizumab-treated (N=10) mice or M81/fLuc infected control (N=6) and Pembrolizumab-treated mice (N=5).

**B95-8**		**Mean**	**SD**	**Mean**	**SD**	**Mean Difference**	**P-value, Mann-Whitney**
**Time point, wpi**	**Group/ Cytokine, pg/ml**	**CTR**	**CTR**	**Pembro (merged)**	**Pembro (merged)**	**CTR vs Pembro (merged)**	**CTR vs Pembro (merged)**
**8**	IFN-γ	1525,04	2137,41	2590,24	3056,14	-1065,00	0,3706
**8**	IL-10	416,73	681,52	825,43	718,53	-408,70	0,2867
**8**	IL-6	367,06	420,30	746,44	627,84	-379,40	0,3706
**8**	IL-8	92,15	82,14	320,46	325,18	-228,30	0,2867
**8**	IL-18	111,19	90,32	208,35	118,12	-97,17	0,2168
**8**	MCP-1	26,64	36,72	79,86	58,56	-53,22	0,1119
**8**	IL-23	152,70	261,52	6,32	6,73	146,40	0,6923
**8**	IL-33	3,90	0,48	10,57	17,06	-6,67	0,1608
**8**	IFN-α	3,07	3,15	11,39	12,09	-8,31	*0,0769*
**8**	IL-17A	1,63	0,93	8,87	9,81	-7,24	***0,049***
**8**	IL-12p70	10,29	16,37	3,56	2,79	6,73	0,5734
**8**	TNF-α	3,88	2,60	5,11	4,01	-1,22	0,5699
**8**	IL-1β	1,89	0,52	2,89	2,59	-1,00	0,8357
**M81**		**Mean**	**SD**	**Mean**	**SD**	**Mean Difference**	**P-value, Mann-Whitney**
**Time point, wpi**	**Group/ Cytokine, pg/ml**	**CTR (PBS+ KIOVIG)**	**CTR (PBS+ KIOVIG)**	**Pembro (merged)**	**Pembro (merged)**	**PBS+ KIOVIG vs Pembro (merged)**	**PBS+ KIOVIG vs Pembro (merged)**
**7,8**	IFN-γ	540,11	497,56	1012,35	1199,50	-472,20	0,7922
**7,8**	IL-10	18,23	10,03	9189,12	11248,73	-9171,00	***0,0173***
**7,8**	IL-6	92,24	90,72	656,10	641,60	-563,90	0,2468
**7,8**	IL-8	97,16	83,60	368,81	390,81	-271,60	0,2468
**7,8**	IL-18	110,42	75,80	104,83	147,45	5,59	0,5368
**7,8**	MCP-1	6,56	7,13	20,04	17,14	-13,48	0,2468
**7,8**	IL-23	6,75	2,06	49,15	51,29	-42,41	*0,0823*
**7,8**	IL-33	3,35	1,03	51,80	70,27	-48,45	***0,0087***
**7,8**	IFN-α	0,97	0,20	25,13	22,94	-24,16	***0,0043***
**7,8**	IL-17A	3,71	1,73	4,07	4,04	-0,35	0,6623
**7,8**	IL-12p70	1,51	0,51	59,21	105,47	-57,70	***0,0043***
**7,8**	TNF-α	3,63	1,99	20,33	22,28	-16,70	0,329
**7,8**	IL-1β	1,72	0,46	3,84	2,12	-2,11	***0,0498***
