# Supplementary material for: PD-1 Blockade Aggravates Epstein–Barr Virus+ Post-Transplant Lymphoproliferative Disorder in Humanized Mice Resulting in Central Nervous System Involvement and CD4+ T Cell Dysregulations
Source: Front Oncol. 2021 Jan 12;10:614876. doi: 10.3389/fonc.2020.614876 (PMC7837057; doi:10.3389/fonc.2020.614876)
Supplement: Supplementary Table 7 — Data presented in Figures 5A, C, E, G and 6A, C, E, G, I, J . Descriptive statistics regarding the B95-8 model for tissue analyses of human immunophenotypic markers measured by flow cytometry. [file Table_7.pdf]

Supplementary Table 7. Data of flow cytometry analyses from organs from B95-8 model. Control (N=3) versus Pembrolizumab-treated mice (N=11).

					**Unpaired Welch's t test**	
**B95-8**	**Mean**	**SD**	**Mean**	**SD**	**Mean Difference**	**P-value**
**Variable**	**CTR**	**CTR**	**Pembro (merged)**	**Pembro (merged)**	**CTR vs Pembro (merged)**	**CTR vs Pembro (merged)**
**Spleen**						
#$CD8^{+}$/$CD45^{+}$ *	6,729	0,2375	7,142	0,5055	0,4132	*0,0802*
#$CD4^{+}$/$CD45^{+}$ *	5,879	0,6688	6,001	0,5949	0,1228	0,7923
%$CD8^{+}$ in$CD45^{+}$	63,3	26,6	57,8	20,4	5,5	0,7620
%$CD4^{+}$ in$CD45^{+}$	10,9	7,3	4,7	2,6	6,2	0,2733
MFI PD1/CD8 *	4,625	0,3375	3,453	0,3307	-1,172	***0,0113***
MFI PD1/CD4 *	4,943	0,2440	3,499	0,2904	-1,443	***0,0013***
%TIM-3^{+} in $CD8^{+}$/$PD1^{-}$	41,4	35,5	64,3	9,8	-22,9	0,3798
%TIM-3^{+} in $CD8^{+}$/$PD1^{+}$	58,3	26,0	79,0	9,7	-20,7	0,3008
%TIM-3^{+} in $CD4^{+}$/$PD1^{-}$	39,8	14,1	45,4	11,8	-5,5	0,5809
%TIM-3^{+} in $CD4^{+}$/$PD1^{+}$	41,0	19,7	57,7	15,4	-16,7	0,2763
%LAG-3^{+} in $CD8^{+}$/$CD69^{-}$	43,2	11,7	56,6	10,0	-13,4	0,1715
%LAG-3^{+} in $CD8^{+}$/$CD69^{+}$	49,1	10,4	61,9	8,9	-12,8	0,1504
%LAG-3^{+} in $CD4^{+}$/$CD69^{-}$	38,6	15,0	51,2	20,0	-12,6	0,2980
%LAG-3^{+} in $CD4^{+}$/$CD69^{+}$	42,3	15,1	55,2	21,0	-13,0	0,2889
%$CD25^{+}$/$FoxP3^{+}$ in $CD4^{+}$	3,1	2,1	7,2	6,5	4,1	0,1045
%$CD45RA^{-}$/$CD25^{+}$/$FoxP3^{+}$ in $CD4^{+}$	2,8	1,8	6,5	5,7	3,7	*0,0876*
%$CD25^{+}$/$FoxP3^{+}$ in $CD45^{-}$/$CD4^{-}$	2,9	0,8	3,7	2,1	0,7	0,3991
%$CD45RA^{-}$/$CD25^{+}$/$FoxP3^{+}$ in $CD45^{-}$/$CD4^{-}$	2,0	0,7	2,8	1,6	0,8	0,2253
**Bone Marrow**						
#$CD8^{+}$/$CD45^{+}$ *	6,265	0,1091	6,133	0,2316	-0,1323	0,1987
#$CD4^{+}$/$CD45^{+}$ *	6,089	0,3541	5,334	0,7523	-0,7542	***0,0401***
%$CD8^{+}$ in$CD45^{+}$	36,3	14,3	49,4	22,4	-13,1	0,2736
%$CD4^{+}$ in$CD45^{+}$	26,1	16,6	15,3	16,3	10,8	0,3861
MFI PD1/CD8 *	5,057	0,4021	4,811	0,5763	-0,2463	0,4379
MFI PD1/CD4 *	4,995	0,04386	3,852	0,3512	-1,143	***<0,0001***
**Lymph node**						
#$CD8^{+}$/$CD45^{+}$ *	3,606	0,4685	4,539	0,6054	0,9334	***0,0450***
#$CD4^{+}$/$CD45^{+}$ *	2,971	0,6431	3,522	1,192	0,5505	0,3255
%$CD8^{+}$ in$CD45^{+}$	28,1	23,6	44,7	28,1	-16,7	0,3604
%$CD4^{+}$ in$CD45^{+}$	9,7	11,7	6,8	7,7	2,9	0,7143
MFI PD1/CD8 *	4,643	0,3147	3,938	0,4080	-0,7047	***0,0316***
MFI PD1/CD4 *	4,752	0,3665	3,937	0,2917	-0,8153	***0,0439***
**Thymus**						
#$CD8^{+}$/$CD45^{+}$ *	4,823	0,4544	5,257	0,3492	0,4342	0,2325
#$CD4^{+}$/$CD45^{+}$ *	5,165	0,3029	5,275	0,3602	0,1098	0,6239
%$CD8^{+}$ in$CD45^{+}$	5,3	1,5	7,6	4,9	-2,3	0,2123
%$CD4^{+}$ in$CD45^{+}$	12,4	6,8	8,6	6,1	3,8	0,4475
MFI PD1/CD8 *	4,354	0,09361	3,745	0,5853	-0,6087	***0,0067***
MFI PD1/CD4 *	4,212	0,2146	3,518	0,4912	-0,6938	***0,0065***

** - original values were log-transformed before statistical tests*
