# Supplementary material for: PD-1 Blockade Aggravates Epstein–Barr Virus+ Post-Transplant Lymphoproliferative Disorder in Humanized Mice Resulting in Central Nervous System Involvement and CD4+ T Cell Dysregulations
Source: Front Oncol. 2021 Jan 12;10:614876. doi: 10.3389/fonc.2020.614876 (PMC7837057; doi:10.3389/fonc.2020.614876)
Supplement: Supplementary Table 8 — Data presented in Figures 5B, D, F, H and 6B, D, F, H, K, L . Descriptive statistics regarding the M81 model for tissue analyses of human immunophenotypic markers measured by flow cytometry. [file Table_8.pdf]

**Supplementary Table 8. Data of flow cytometry analyses from organs for M81 model.** Control (N=6) versus Pembrolizumab-treated mice (N=5, SPL, BM, Thy; N=3, LN).

| | | | | | Unpaired Welch's t test | |
|---|---|---|---|---|---|---|
| **M81** | **Mean** | **SD** | **Mean** | **SD** | **Mean Difference** | **P-value** |
| **Variable** | **CTR** | **CTR** | **Pembro (merged)** | **Pembro (merged)** | **CTR vs Pembro (merged)** | **CTR vs Pembro (merged)** |
| **Spleen** | | | | | | |
| #CD8$^+$/CD45$^+$ | 7,082 | 0,2718 | 6,552 | 0,3818 | -0,5293 | ***0,0351*** |
| #CD4$^+$/CD45$^+$ | 6,927 | 0,3116 | 6,018 | 0,3666 | -0,9085 | ***0,0024*** |
| %CD8$^+$ inCD45$^+$ | 50,1 | 4,9 | 32,1 | 27,8 | 17,9 | 0,2235 |
| %CD4$^+$ inCD45$^+$ | 35,3 | 5,9 | 8,3 | 5,2 | 27 | ***<0,0001*** |
| MFI PD1/CD8 | 4,996 | 0,08639 | 3,875 | 0,7680 | -1,121 | ***0,0305*** |
| MFI PD1/CD4 | 5,154 | 0,0617 | 3,987 | 0,5894 | -1,167 | ***0,0112*** |
| %TIM-3$^+$ in CD8$^+$/PD1$^-$ | 1,3 | 0,9 | 23,4 | 29,9 | -22,2 | 0,1727 |
| %TIM-3$^+$ in CD8$^+$/PD1$^+$ | 26,2 | 10,0 | 36,7 | 28,6 | -10,5 | 0,4722 |
| %TIM-3$^+$ in CD4$^+$/PD1$^-$ | 0,6 | 0,2 | 12,5 | 12,7 | -11,9 | 0,1038 |
| %TIM-3$^+$ in CD4$^+$/PD1$^+$ | 13,1 | 5,4 | 32,3 | 21,8 | -19,2 | 0,1194 |
| %LAG-3$^+$ in CD8$^+$/CD69$^-$ | 19,1 | 2,6 | 15,5 | 4,5 | 3,6 | 0,1602 |
| %LAG-3$^+$ in CD8$^+$/CD69$^+$ | 21,7 | 7,1 | 57,9 | 20,9 | -36,2 | ***0,0152*** |
| %LAG-3$^+$ in CD4$^+$/CD69$^-$ | 14,8 | 2,9 | 12,1 | 6,8 | 2,7 | 0,4493 |
| %LAG-3$^+$ in CD4$^+$/CD69$^+$ | 18,8 | 6,1 | 57,6 | 27,5 | -38,8 | ***0,0329*** |
| %CD25$^+$/FoxP3$^+$ in CD4$^+$ | 3,1 | 0,764 | 20,1 | 14,7 | -17,0 | *0,0609* |
| %CD45RA$^-$/CD25$^+$/FoxP3$^+$ in CD4$^+$ | 2,8 | 0,6 | 10,3 | 5,7 | 7,6 | ***0,0407*** |
| %CD25$^+$/FoxP3$^+$ in CD45$^-$/CD4$^-$ | 0,4 | 0,2 | 6,4 | 5,9 | 6,0 | *0,0868* |
| %CD45RA$^-$/CD25$^+$/FoxP3$^+$ in CD45$^-$/CD4$^-$ | 0,2 | 0,07 | 3,6 | 3,2 | 3,3 | *0,0847* |
| **Bone Marrow** | | | | | | |
| #CD8$^+$/CD45$^+$ | 6,305 | 0,1993 | 6,196 | 0,1644 | -0,1088 | 0,4144 |
| #CD4$^+$/CD45$^+$ | 6,230 | 0,2079 | 5,461 | 0,5021 | -0,7695 | ***0,0231*** |
| %CD8$^+$ inCD45$^+$ | 31,77 | 8,40 | 29,47 | 21,41 | 2,3 | 0,8302 |
| %CD4$^+$ inCD45$^+$ | 26,07 | 5,95 | 9,77 | 9,77 | 16,3 | ***0,0159*** |
| MFI PD1/CD8 | 3,947 | 0,07807 | 3,421 | 0,6368 | -0,5252 | 0,1390 |
| MFI PD1/CD4 | 4,139 | 0,06208 | 3,726 | 0,3690 | -0,4132 | *0,0658* |
| **Lymph node** | | | | | | |
| #CD8$^+$/CD45$^+$ | 5,225 | 0,3482 | 4,837 | 1,534 | -0,3872 | 0,7819 |
| #CD4$^+$/CD45$^+$ | 5,122 | 0,4682 | 4,652 | 2,117 | -0,4701 | 0,8065 |
| %CD8$^+$ inCD45$^+$ | 46,6 | 13,8 | 25,1 | 11,9 | 21,5 | *0,0634* |
| %CD4$^+$ inCD45$^+$ | 37,7 | 13,0 | 20,2 | 25,8 | 17,5 | 0,3622 |
| MFI PD1/CD8 | 4,982 | 0,07252 | 3,990 | 0,2084 | -0,9912 | ***0,0108*** |
| MFI PD1/CD4 | 5,180 | 0,0523 | 3,932 | 0,2135 | -1,249 | ***0,0081*** |
| **Thymus** | | | | | | |
| #CD8$^+$/CD45$^+$ | 4,947 | 0,2685 | 5,009 | 0,3838 | 0,06199 | 0,7697 |
| #CD4$^+$/CD45$^+$ | 5,403 | 0,3096 | 5,429 | 0,2930 | 0,02571 | 0,8909 |
| %CD8$^+$ inCD45$^+$ | 3,6 | 1,2 | 6,3 | 4,7 | -2,66 | 0,2800 |
| %CD4$^+$ inCD45$^+$ | 11,3 | 7,7 | 17,4 | 16,1 | -6,17 | 0,4640 |
| MFI PD1/CD8 | 4,679 | 0,1004 | 4,160 | 0,3787 | -0,5185 | ***0,0356*** |
| MFI PD1/CD4 | 4,257 | 0,06919 | 3,982 | 0,3238 | -0,2750 | 0,1307 |

** - original values were log-transformed before statistical tests*
