# Supplementary material for: PD-1 Blockade Aggravates Epstein–Barr Virus+ Post-Transplant Lymphoproliferative Disorder in Humanized Mice Resulting in Central Nervous System Involvement and CD4+ T Cell Dysregulations
Source: Front Oncol. 2021 Jan 12;10:614876. doi: 10.3389/fonc.2020.614876 (PMC7837057; doi:10.3389/fonc.2020.614876)
Supplement: Supplementary Table 9 — Data presented in Figures 7D–I . Descriptive statistics regarding tissue multiplex IHC analyses of spleen and liver tissues explanted from B95-8-infected mice. [file Table_9.pdf]

Supplementary Table 9. Tissue multiplex IHC analyses. Data shown for control versus Pembrolizumab-treated mice in B95-8/fLuc model.

			Unpaired Welch's t test			
**B95-8**	**Mean**	**SD**	**Mean**	**SD**	**Mean Difference**	**P-value**
**Tissue/ Marker**	**PBS**	**PBS**	**Pembro (merged)**	**Pembro (merged)**	**CTR vs Pembro (merged)**	**CTR vs Pembro (merged)**
**Liver**	**Control (n=3) and Pembrolizumab-treated (n=11)**					
**Parenchyma**						
#$CD4^+$/$Ki67^+$/mm^2	31,4	20,52	19,13	11,16	12,26	0,4111
#$CD8^+$/$Ki67^+$/mm^2	207,3	143,1	457,6	324,1	-250,4	*0,0849*
#$CD4^+$/$FoxP3^+$/mm^2	1,651	0,564	1,224	1,207	0,4267	0,4085
#$CD8^+$/$FoxP3^+$/mm^2	0	0	0,285	0,477	-0,2854	0,0755
**Tumor**						
#$CD4^+$/$Ki67^+$/mm^2	325,7	278,7	49,69	65,19	276,1	0,2270
#$CD8^+$/$Ki67^+$/mm^2	1412,0	141,4	1848	1049	-436,3	0,2084
#$CD4^+$/$FoxP3^+$/mm^2	0,23	0,407	5,617	9,449	-5,382	0,0887
#$CD8^+$/$FoxP3^+$/mm^2	11,16	19,33	2,589	4,837	8,569	*0,5236*
#$CD4^+$/$FoxP3^+$ Ø CD4	0,016	0,027	0,352	0,400	-0,336	***0,0193***
#$CD8^+$/$FoxP3^+$ Ø CD4	0,016	0,028	0,103	0,210	-0,087	0,2092
#$CD4^+$/$FoxP3^+$ Ø CD8	0,009	0,016	0,249	0,302	-0,24	***0,0251***
#$CD8^+$/$FoxP3^+$ Ø CD8	0,004	0,007	0,120	0,177	-0,116	*0,0558*
**Spleen**	**Control (n=2) and Pembrolizumab-treated (n=11)**					
**Parenchyma**						
#$CD4^+$/$Ki67^+$/mm^2	284,2	366,6	204,2	251,9	80,03	0,8107
#$CD8^+$/$Ki67^+$/mm^2	2801	2291	3558	1303	-757	0,7219
#$CD4^+$/$FoxP3^+$/mm^2	21,25	30,06	15,85	26,06	5,404	0,8438
#$CD8^+$/$FoxP3^+$/mm^2	4,375	3,663	9,556	12,41	-5,181	0,2944
**Tumor**						
#$CD4^+$/$Ki67^+$/mm^2	259,8	362,8	86,66	135,4	173,2	0,6212
#$CD8^+$/$Ki67^+$/mm^2	927,8	121	1849	891,2	-921,1	***0,0075***
#$CD4^+$/$FoxP3^+$/mm^2	15,92	22,51	8,539	15,36	7,38	0,7243
#$CD8^+$/$FoxP3^+$/mm^2	3,106	4,393	6,654	11,25	-3,548	0,4814
