# Supplementary material for: PD-1 Blockade Aggravates Epstein–Barr Virus+ Post-Transplant Lymphoproliferative Disorder in Humanized Mice Resulting in Central Nervous System Involvement and CD4+ T Cell Dysregulations
Source: Front Oncol. 2021 Jan 12;10:614876. doi: 10.3389/fonc.2020.614876 (PMC7837057; doi:10.3389/fonc.2020.614876)
Supplement: Supplementary Table 10 — Antibodies used for flow cytometry and histopathology analyses. [file Table_10.pdf]

Supplementary Table 10. Antibodies used in the study:

Antigen	Detection	Vendor	Dilution	Catalogue #
**CD279 (PD-1)**	PE	Biolegend	1:100	329906
**CD3**	BV510	Biolegend	1:100	300448
**CD4**	PerCP	Biolegend	1:250	317432
**CD4**	AH7	BD	1:100	560158
**CD45**	Pacific Blue	Biolegend	1:100	304022
**CD45RA**	FITC	Beckman Coulter	1:100	A07786
**CD8a**<br>**CD8a**	PE-Cy7<br>FITC	Biolegend<br>Biolegend	1:100	300914
**CD19**	AL700	Biolegend	1:250	302226
**FoxP3**	PE	BD	1:50	560046
**CD25**	APC	Biolegend	1:100	101909
**LAG3**	PE-Cy5	eBio	1:100	317444
**TIM3**	APC	eBio	1:100	345012
**CD69**	APC	Biolegend	1:100	310910
**<u>Histopathology</u>**<br>**CD4**	Opal520	ZYTOMED	1:50	503-3354
**CD8**	Opal570	Dako	1:600	M7103
**FoxP3**	Opal620	Abcam	1:200	Ab20034
**CD20**	Opal650	Dako	1:500	M0755
**Ki67**	Opal690	Thermo scientific	1:200	RM9106-S1
**PDL1**	DAB	Quartett	1:100	1/PR2992/07
**CD30**	DAB	Dako	1:50	M0751
**CD8**	DAB	Dako	1:100	M7103
**CD3**	DAB	Dako	1:100	A0452
**Anti-human IgG**	HRP	Roth	1:500	47531
**Anti-mouse IgG**	AL647	Biolegend	1:250	405322
**Anti-mouse IgG**	AL488	Biolegend	1:250	405319
**CD3**	DAB	Dako	1:50	M7254
**Anti-mouse IgG**	Biotin-SP	Jackson	1:200	115-065-207
